# A microsatellite repeat in *PCA3* long non-coding RNA is associated with prostate cancer risk and aggressiveness

**DOI:** 10.1038/s41598-017-16700-y

**Published:** 2017-12-04

**Authors:** John Lai, Leire Moya, Jiyuan An, Andrea Hoffman, Srilakshmi Srinivasan, Janaththani Panchadsaram, Carina Walpole, Joanna L. Perry-Keene, Suzanne Chambers, Australian Prostate Cancer BioResource, T. Yeadon, P. Saunders, A. Eckert, P. Heathcote, G. Wood, G. Malone, H. Samaratunga, A. Collins, M. Turner, K. Kerr, Melanie L. Lehman, Colleen C. Nelson, Judith A. Clements, Jyotsna Batra

**Affiliations:** 1 Australian Prostate Cancer Research Centre – Queensland, Translational Research Institute, Brisbane, 4102 Australia; 2 Cancer Program, School of Biomedical Sciences, Institute of Health and Biomedical Innovation, Queensland University of Technology, Brisbane, 4102 Australia; 3 Anatomical Pathology, Pathology Queensland, Brisbane, Australia; 4 Allied Health Research, Menzies Health Institute Queensland, Griffith University, Brisbane, 4111 Australia; 5 Brisbane Urology Clinic, Brisbane, 4000 Australia; 6 Aquesta Uropathology, Brisbane, 4066 Australia; 7 Sullivan and Nicolaides Pathology, Brisbane, 4006 Australia

## Abstract

Short tandem repeats (STRs) are repetitive sequences of a polymorphic stretch of two to six nucleotides. We hypothesized that STRs are associated with prostate cancer development and/or progression. We undertook RNA sequencing analysis of prostate tumors and adjacent non-malignant cells to identify polymorphic STRs that are readily expressed in these cells. Most of the expressed STRs in the clinical samples mapped to intronic and intergenic DNA. Our analysis indicated that three of these STRs (TAAA-*ACTG2*, TTTTG-*TRIB1*, and TG-*PCA3*) are polymorphic and differentially expressed in prostate tumors compared to adjacent non-malignant cells. TG-*PCA3* STR expression was repressed by the anti-androgen drug enzalutamide in prostate cancer cells. Genetic analysis of prostate cancer patients and healthy controls (N > 2,000) showed a significant association of the most common 11 repeat allele of TG-*PCA3* STR with prostate cancer risk (OR = 1.49; 95% CI 1.11–1.99; *P* = 0.008). A significant association was also observed with aggressive disease (OR = 2.00; 95% CI 1.06–3.76; *P* = 0.031) and high mortality rates (HR = 3.0; 95% CI 1.03–8.77; *P* = 0.045). We propose that TG-*PCA3* STR has both diagnostic and prognostic potential for prostate cancer. We provided a proof of concept to be applied to other RNA sequencing datasets to identify disease-associated STRs for future clinical exploratory studies.

## Introduction

Short tandem repeats (STRs) are repetitive sequences of two to six nucleotides in a genome. Polymorphic STRs resulting from STR expansion or contraction is thought to result from replication slippage^1,2^. STRs are generally highly polymorphic and widely distributed in the human genome^3^. These features have resulted in their widespread use as genetic markers in genealogy and forensic science^4^. Further, there is compelling evidence to indicate that the expansion of STRs within genes can cause disease whereby recent studies have shown their correlation with gene expression^5,6^. For example, the first report of an STR causing a disease was a CAG expansion in exon 1 of the androgen receptor (AR) gene that leads to spino-bulbular muscular atrophy^7^. Since then, the expansion of STRs has been implicated in over 40 other Mendelian diseases^8^, with many of these conditions catalogued in an online database^9^. Notably, CAG STRs are commonly found in regulatory proteins, and the expansion of these CAG repeats within these genes affects protein functionality^10^. Interestingly, it has been proposed that the actual repetitive protein sequence (encoded by the STR) is what is ultimately most important in causing poly-glutamine diseases such as Huntington’s disease^11^. There is a growing interest in STRs as modulators of disease^12^, with recent concerted efforts being made in characterizing STRs in the human genome using high-throughput DNA sequencing approaches^3,13–15^. The role of STR polymorphisms in prostate cancer is less known, with many of the studies focusing on the exon 1 CAG and GGN repeats in the AR^16–20^. For example, a meta-analysis of earlier genetic association studies suggest that a lesser number of CAG and GGN repeats in the AR confers increased risk for prostate cancer^16^. Indeed, functional promoter reporter assays indicate that shorter ARs resulting from CAG contraction increases the AR’s ability to activate genes^21^.

Genome wide association studies (GWAS) using single nucleotide polymorphisms (SNPs) have identified ~100 regions in the human genome that confer prostate cancer risk^22^. Despite the advances made by GWAS, SNPs only account for ~33% of familial prostate cancers^22^. This indicates that the majority (up to 67%) of heritable prostate cancer risk lies in other types of genetic variation. Thus, this study focuses on the potential of STRs to account for some of the ‘missing heritability’ of prostate cancer given the aforementioned characteristics of STRs. Here, we investigate STRs in prostate cancer RNAseq datasets to direct us to polymorphic STRs that have potential utility as risk indicators for prostate cancer risk and/or prognosis. A TG dinucleotide repeat in *PCA3* was significantly associated with prostate cancer risk and aggressiveness in our analysis of over 2,000 prostate cancer patients and controls.

## Results

### STRs in the human genome are predicted to be polymorphic and are widely distributed

**Figure 1:**
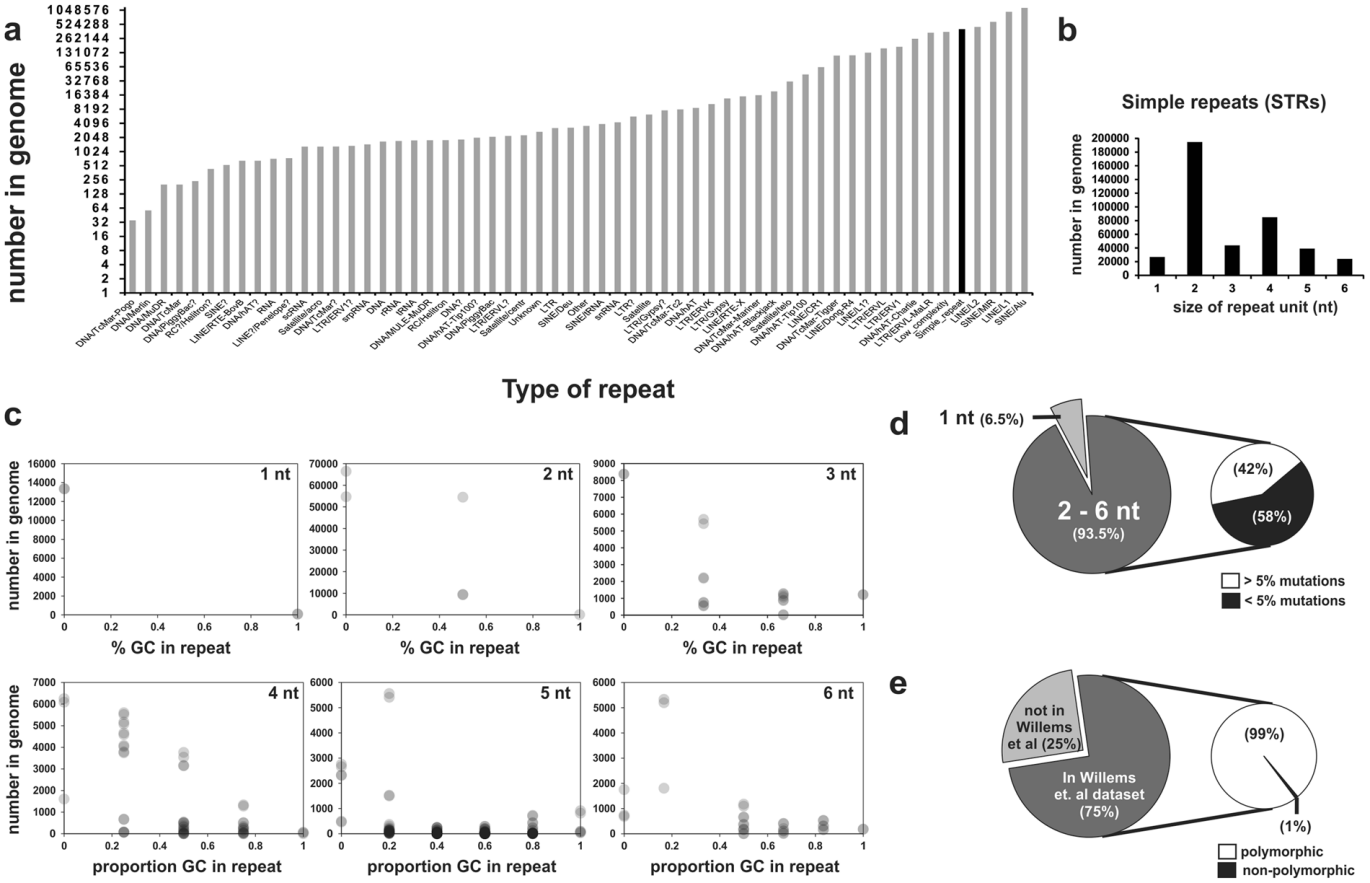
Characterisation of STRs in the human genome. (**a**) Histogram of the total number of repetitive units in the genome that includes 413,414 STRs (Simple_repeats, black bar) from the Repeat Masker library. **(b**) Histogram indicating that the genome mostly comprises di-nucleotide repeats, and that hexa-nucleotide repeats occur in the least amount. **(c**) Scatterplot indicating that the genome comprises mostly of STRs with low numbers of G and C nucleotides (% GC in repeat). **(d**) Pie charts indicating that of the STRs that comprise of 2–6 nt nucleotides, 223,742 STRs (58%) have less than 5% mutations, insertions or deletions. **(e**) Pie charts indicating that 121,835 of the 223,742 STRs (75%) from the Repeat Masker library were detected in the Willems *et al*. Phase 1, 1000 genome dataset^3^. 120,806 of these STRs (99%) were predicted by the Willems *et al*. study to be polymorphic.

An analysis was undertaken to assess the occurrence of STRs within the human genome in order to determine whether they have potential as a genetic marker for prostate cancer risk. Figure 1a indicates that there are 413,414 STRs (Simple_repeats in the RepeatMasker library) in the human genome, and that STRs are the fifth most frequently found repetitive motif.

The four most frequent types of repetitive DNA are from LINEs and SINEs. SNPs within these LINEs and SINEs have already been studied in GWAS. Thus, STRs represent an understudied reservoir of an alternative genetic variation for genetic epidemiology studies. Most of these STRs are di-nucleotide repeats (Fig. 1b). Notably, there are far fewer STRs that have a GC nucleotide composition over 50% (Fig. 1c). This indicates that the human genome selects against STRs with G and C nucleotides, and that the composition of STRs within genes can potentially affect biology.

Mono-repetitive STRs were excluded from further analysis as current fragment analysis platforms using capillary separation are not able to accurately resolve one nucleotide differences in STR alleles. This resulted in the exclusion of 26,872 STRs (6.5%) for further analysis (Fig. 1d). STRs with over 5% mutation/deletion/insertion were also excluded from further analysis as the focus of this study is on the expansion of STRs which might affect prostate cancer, rather than sequence transitions. This filtering resulted in the exclusion of 162,800 STRs (42%), leaving 223,742 STRs (58%) for further analysis (Fig. 1d).

An analysis was then performed to determine which of these 223,742 STRs are polymorphic. Thus, these STRs were screened against the Willems *et al*. dataset of (non)-polymorphic STRs that were previously analyzed on the Phase 1, 1000 Genome Project datasets^3^. Using a custom Perl script, 121,835 STRs (75%) were detected in the Willems *et al*. dataset, and of these, 120,806 STRs (99%) were predicted by the Willems *et. al*. study to be polymorphic (Fig. 1e). The custom Perl script was used to identify STRs that were detected in both the RepeatMasker dataset and the Willems *et al*. dataset to ensure that high-confidence STRs were selected for in this study. This conservative filtering process provided us with a strong list of putative polymorphic STRs to interrogate in prostate cancer RNAseq datasets.

### STRs are readily expressed in prostate cancer cells

**Figure 2:**
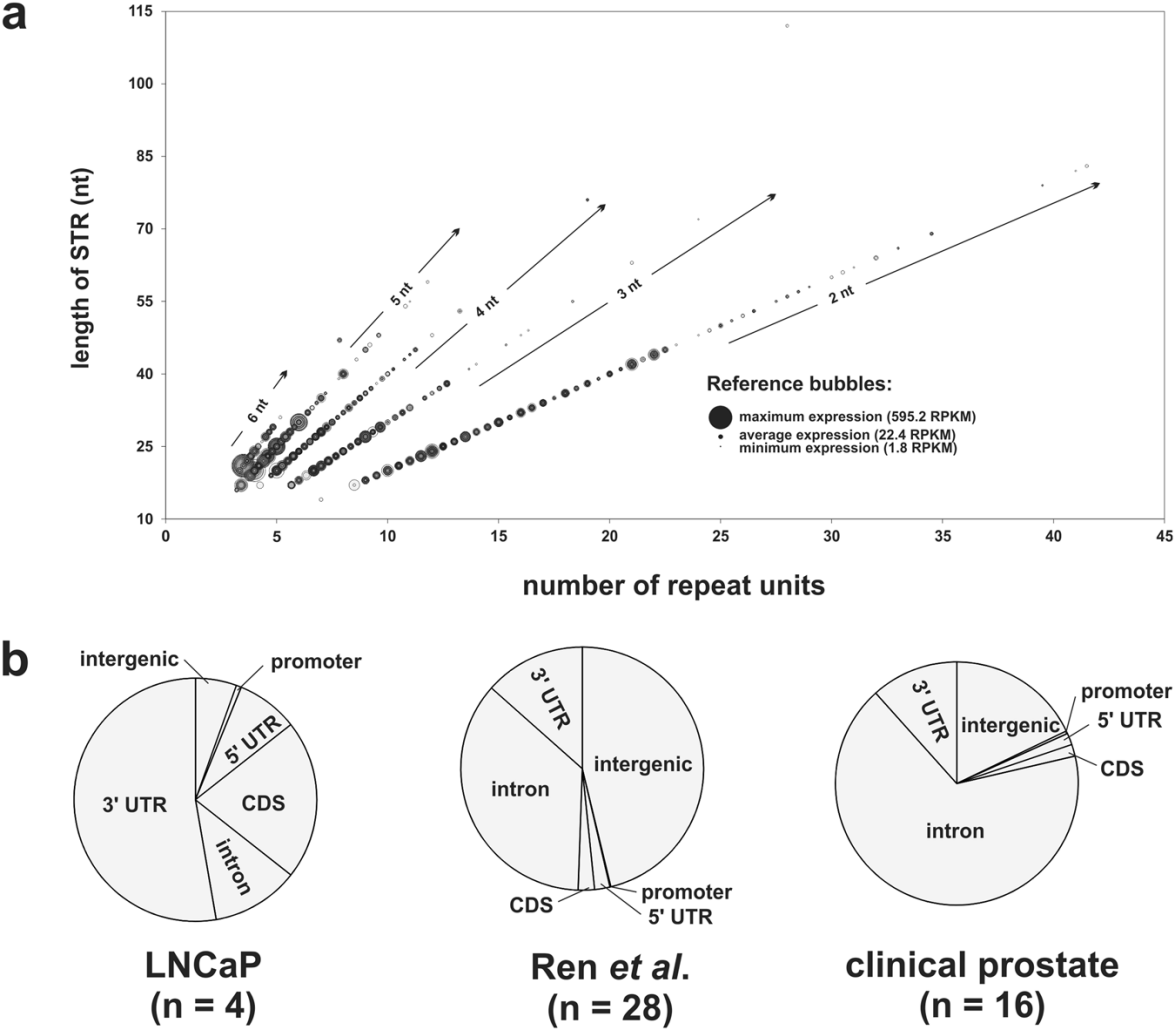
STR expression in RNAseq datasets. (**a**) Bubble plot of STR expression for di-(2 nt), tri- (3 nt), tetra- (4 nt), penta- (5 nt) and hexa- (6 nt) nucleotide repeats. Larger sized bubbles indicate higher expression for that respective STR. Darker intensity bubbles indicate that multiple STRs of that particular length and respective number of repeat unit are expressed. **(b**) Pie chart detailing the percentage of expressed STRs that are located within intergenic, promoter, 5′UTR, coding (CDS), intronic, or 3′UTR DNA in LNCaP cells, the Ren et al. clinical prostate cancer RNAseq dataset^40^, and our eight clinical prostate samples.

Figure 2a and Supplementary Figure S1 shows the bubble plots of STR expression in LNCaP prostate cancer cells that were treated with androgen (DHT) or therapeutic anti-androgens (bicalutamide, enzalutamide), and in clinical prostate cancer tissue and their corresponding adjacent non-cancer prostate cells.

A larger circle indicates that a particular STR is more highly expressed compared to other STRs (smaller circles). Figure 2a indicates that transcripts tend to select against longer STRs with a consequent high number of repeats, and that this is consistent for di-, tri-, tetra-, penta-, and hexa-nucleotide repeat STRs. Notably, the more highly expressed STRs are comprised of penta- and hexa-nucleotide repeats, and the location of STR expression within the genome differed between the LNCaP cell line and clinical samples. For example, the majority of expressed STRs in LNCaP cells are located within 3′UTRs and coding DNA, whereas expressed STRs from the clinical samples were predominantly located in intronic and intergenic DNA (Fig. 2b and Supplementary Table S1).

### STRs are differentially expressed in prostate tumors relative to adjacent non-cancer cells

**Figure 3:**
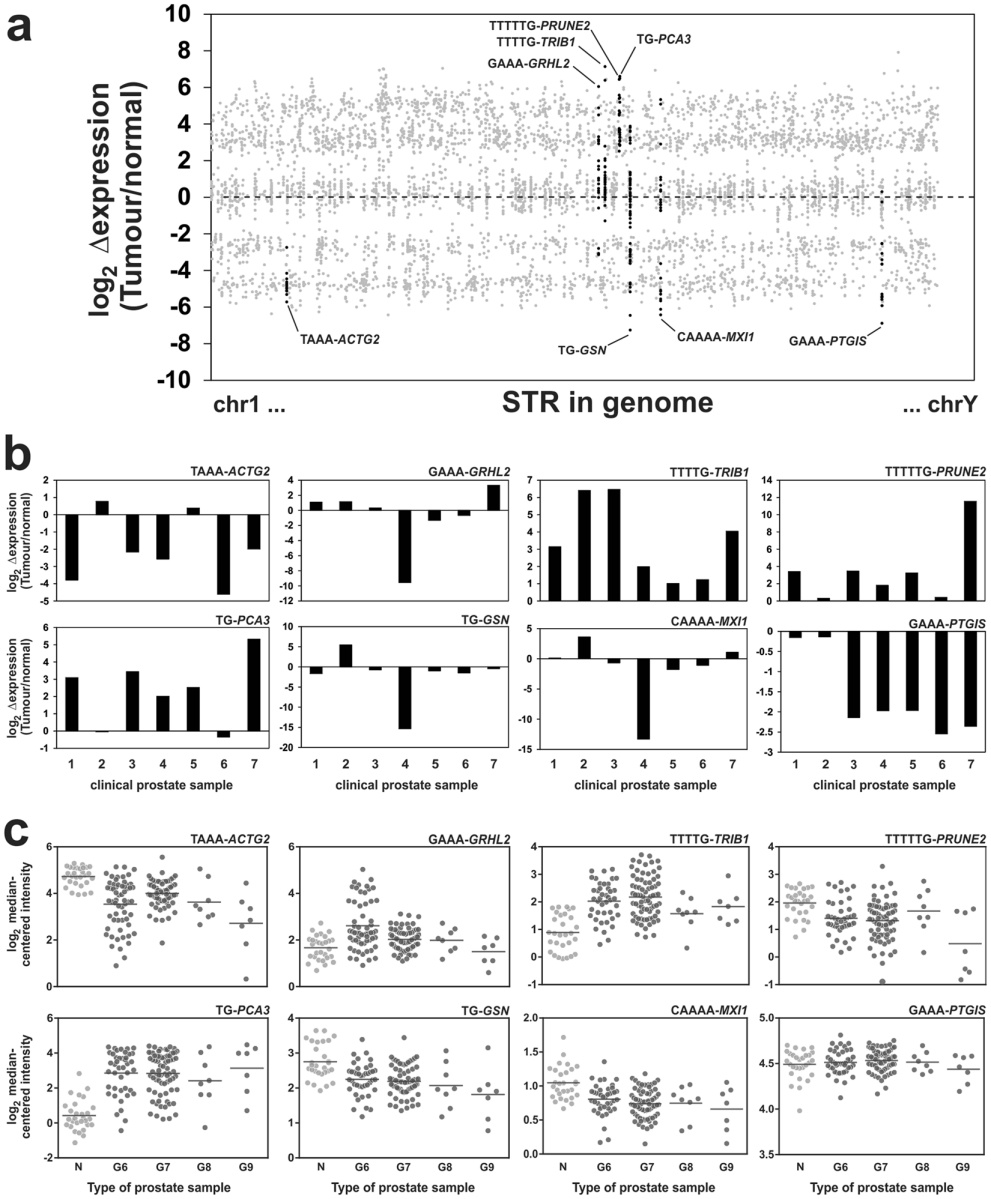
Scatterplot of differential STR expression between tumors and adjacent non-cancer prostate cells. **(a**) Highlighted in black dots are 8 candidate STRs that are consistently differentially expressed in RNAseq datasets, and/or are expressed in a large number of RNAseq datasets from ours (n = 8) and Ren *et al*. (n = 14) clinical prostate samples^40^. **(b**) RT-qPCR analysis of the 8 candidate STRs in another cohort (n = 7) of clinical prostate samples. **(c**) Analysis of microarray expression data from the Taylor *et al*. study in non-cancer cells (N), and prostate cancers of Gleason score 6–9 (G6, G7, G8, and G9). The horizontal line represents the mean expression for each group.

STRs from the RNAseq analysis were prioritized for candidate level validation based on whether they are frequently expressed, and whether they are differentially expressed in prostate tumors relative to their adjacent non-malignant prostate cells. Thus, a metric was developed that consisted of the sum of the fold change in STR expression (tumor ÷ non-cancer) for each STR multiplied by the number of samples that expressed that particular STR. This enabled the detection of STRs that are consistently differentially expressed in tumors, and/or which are readily expressed in multiple tumors (Supplementary Figure S2). From this sorted list, four STRs that had the highest value (over-expressed in tumors), and four STRs with the lowest value (under-expressed in tumors) were prioritized for further analysis (Fig. 3a).

These STRs were located within the actin, gamma 2, smooth muscle, enteric (*ACTG2*), grainyhead-like 2 (Drosophila) (*GRHL2*), tribbles pseudokinase 1 (*TRIB1*), prune homolog 2 (Drosophila) (*PRUNE2*), prostate cancer associated 3 (non-protein coding) (*PCA3*), gelsolin (*GSN*), MAX interactor 1, dimerization protein (*MXI1*), and prostaglandin I2 (prostacyclin) synthase (*PTGIS*) genes (Supplementary Table S2.).

RT-qPCR analysis of seven additional clinical prostate tumors and their adjacent non-malignant prostate cells confirmed that TAAA-*ACTG2* (5/7 cases), GAAA-*PTGIS* (5/7 cases) are consistently down-regulated (>2-fold change in expression) in tumors compared to adjacent non-malignant tissues, and that TTTTG-*TRIB1* (7/7 cases), TTTTTG-*PRUNE2* (5/7 cases), TG-*PCA3* (5/7 cases) are consistently up-regulated (>2-fold change in expression) in tumors compared to adjacent non-malignant cells (Fig. 3b and Table 1).

**Table 1:** Summary of eight candidate STRs.

STR	Loci^a^	Tumor expression^b^	(anti)-androgen regulation^c^ in LNCaP cells	Alleles
TAAA-*ACTG2*	chr2:74144316–74144336	Down	Not expressed	5, 6
GAAA-*GRHL2*	chr8:102563848–102563874	No change	Not regulated	4, 5
TTTTG-*TRIB1*	chr8:126450287–126450311	Up	DHT (↓)	3, 4, 5
TTTTTG-*PRUNE2*	chr9:79395653–79395679	Up	Not assessed	Not polymorphic
TG-*PCA3*	chr9:79400650–79400676	Up	Enzalutamide (↓)	9, 10, 11, 12, 13
TG-*GSN*	chr9:124094978–124094997	No change	Not assessed	Not genotyped
CAAAA-*MXI1*	chr10:112044843–112044867	No change	Enzalutamide (↓), DHT (↓)	4, 5
GAAA-*PTGIS*	chr20:48121708–48121728	Down	Not assessed	Not polymorphic

^a^Repeat Masker coordinate (hg19). ^b^RT-qPCR validated expression in at least four of seven tumors with over 2-fold change in expression. ^c↓^Indicates down-regulation by the respective (anti)-androgen. STR loci locations, their respective expression in tumor and in (anti)-androgen LNCaP cells and predicted number of repeats.

Notably, apart from TTTTTG-*PRUNE2*, edgeR analysis indicates that these eight STRs have similar expression profiles as the genes that they are located in (Supplementary Figure S3). The gene expression profile of the prostate cancer biomarker alpha-methylacyl-CoA racemase (*AMACR*) in these seven clinical samples was used as a positive control (Supplementary Figure S4). The predicted number of alleles for each STR is indicated in Supplementary Table S2.

Further examination of differential expression of the genes that harbor the eight candidate STRs using the Taylor *et al*. microarray study^23^ confirmed our observations that *TRIB1*, *PCA3*, and *GRHL2* are over-expressed in prostate cancer, and that *ACTG2*, *GSN*, and *MXI1* are down-regulated in prostate cancers (Fig. 3c). Notably, our analysis of the Taylor *et al*. data sets showed no significant differences in expression for *PTGIS* only (*P = *0.36, Supplementary Table S3) where expression was found to be under expressed in prostate cancers compared to adjacent non-cancer cells.

### STRs are regulated by androgens and/or therapeutic anti-androgens

**Figure 4:**
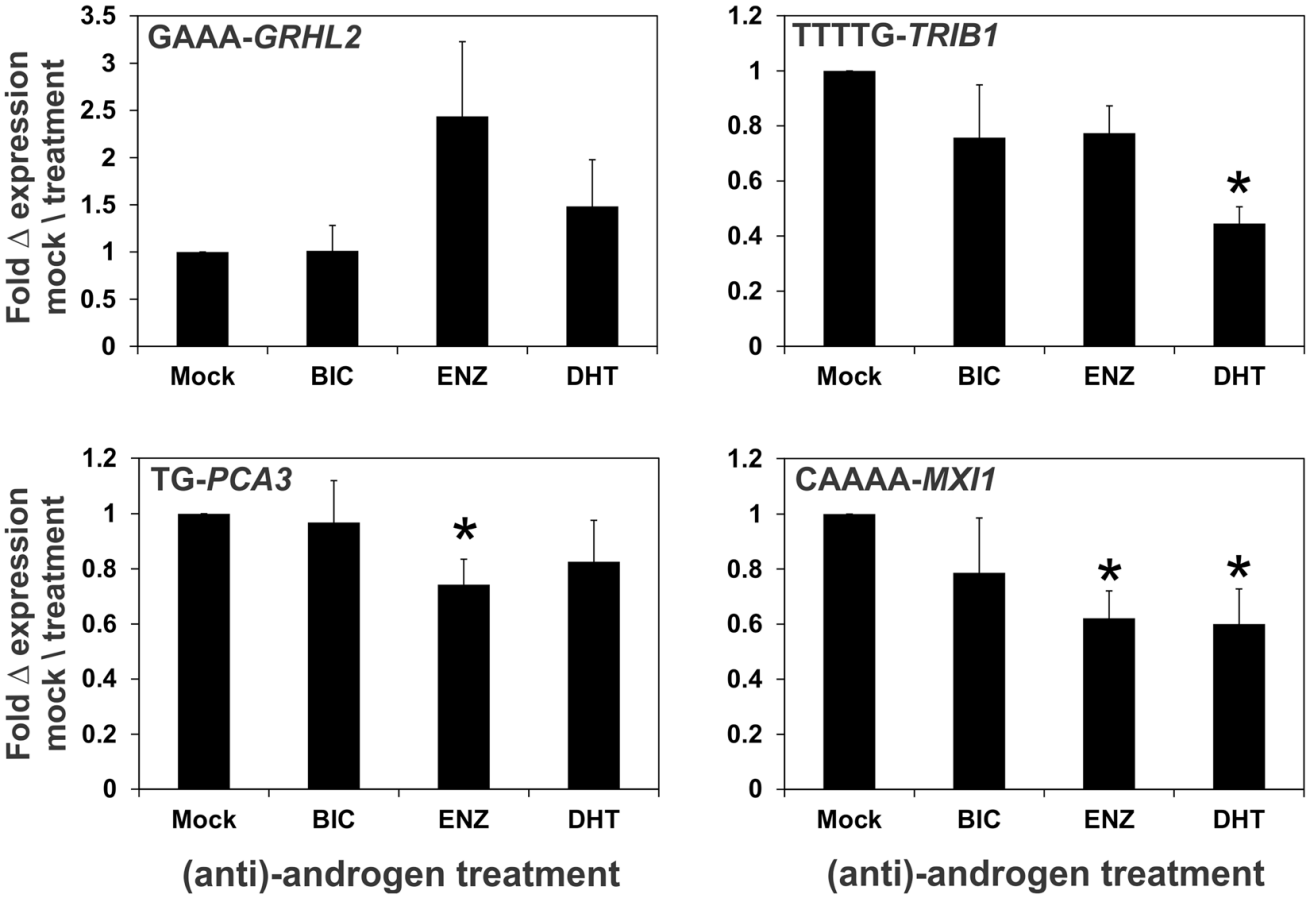
(Anti)-androgen regulation of STRs in LNCaP prostate cancer cells. LNCaP cells were treated with either ethanol (Mock), 10 μM anti-androgens (bicalutamide (BIC), enzalutamide (ENZ)), or 10 nM androgen (DHT) for 24 h. Data is represented as the SEM from 6 independent RNA. The * denotes a significant (*P* < 0.05) difference in expression relative to Mock treated cells.

An RT-qPCR analysis of the androgen and anti-androgen regulation of the five candidate STRs was performed given the importance of the AR signaling pathway in prostate cancer progression. Of the five STRs, only TAAA-*ACTG2* was not expressed in LNCaP prostate cancer cells (Table 1). Our expression analysis revealed that TTTTG-*TRIB1* and CAAAA-*MXI1* are down-regulated by androgen (DHT), while TG-*PCA3* and CAAAA-*MXI1* are down-regulated by the therapeutic anti-androgen, enzalutamide (ENZ) in LNCaP cells (Fig. 4 and Table 1). The expression of the prototypical androgen-regulated *KLK3* gene was used to ensure that cells were appropriately treated (Supplementary Figure S4).

### TG*-PCA3* is associated with prostate cancer risk

An analysis of 40 men with prostate cancer indicated that TG-*GSN* could not be accurately genotyped, and that TTTTTG-*PRUNE2* and GAAA-*PTGIS* are not polymorphic (Supplementary Table S4.). All five candidate polymorphic STRs were within Hardy-Weinberg Equilibrium and have a heterozygosity index between 0.05–0.575 (Supplementary Table S4.).

Since *PCA3* is an emerging biomarker for prostate cancer, and that the expression of the TG STR in *PCA3* gene is regulated by anti-androgen therapy in this study, we selected the TG-*PCA3* STR for a genetic association analysis in a large cohort of prostate cancer patients and controls. Table 2 illustrates a number of the socio-demographic and clinical characteristics of the sample set in our study. There were no significant differences in the BMI and other factors of the patient and control group. However, a significant difference was observed between the mean age between the two groups (*P* < 0.0001). A total of 68 patients had all-cause mortality, while only 24 patients had prostate cancer specific mortality.

**Table 2:** Socio-demographic and clinical characteristics of the QLD study populations.

Characteristics	Men with prostate cancer (*n* = 1,153) *n* (%)	Healthy controls (*n* = 1,210) *n* (%)	*P* values
Age in years (median, range)	63.1 (42.6–87.1)	61.8 (18–90)	*P* < 0.0001^c^
BMI (Mean, SD)	28.4 (4.7)	27.9 (4.5)	*P* = 0.08^c^
**Marital status**			
Never married	46 (4)	88 (8)	*P* = 0.17^c^
Married/de facto	931 (85)	952 (81)	
Divorced/separated/widowed	117 (11)	133 (11)	
Unknown	59 (5)*	37 (3)*	
**Family history of prostate cancer** ^**a**^			
No	499 (66)	807 (90)	*P* > 0.9^d^
Yes	262 (34)	94 (10)	
Unknown	392 (34)*	309 (25)*	
**Vasectomy status** ^**b**^			
No	283 (66)	709 (61)	*P* > 0.9^d^
Yes	146 (34)	447 (39)	
Unknown	724 (63)*	54 (4)*	
**Smoking status**			
Never smoked	418 (38)	500 (43)	*P* = 0.17^a^
Former smoker	589 (54)	591 (50)	
Current smoker	80 (7)	81 (7)	
Unknown	66 (6)*	38 (3)*	
**Alcohol consumption** ^**b**^			
Non-drinker	61 (14)	151 (13)	*P* > 0.9^d^
Drinker	367 (86)	1021 (87)	
Unknown	725 (63)*	38 (3)*	
**Highest education level achieved**			
No formal education	10 (1)	16 (1)	*P* = 0.99^c^
Primary/Secondary school	513 (47)	471 (40)	
Professional qualification	355 (33)	374 (32)	
University degree	212 (19)	311 (27)	
Unknown	63 (6)*	38 (3)*	
**Gleason score (Gleason grade 1** + **Gleason grade 2)**			
<8	916 (79)	Not applicable	
≥8	145 (13)	Not applicable	
Unknown	92 (8)	Not applicable	

^a^Positive family history is defined as at least one first degree relative with prostate cancer. ^b^Data was not collected for the retrospective study. *(%) with respect to the whole cohort. Individuals with “unknown” characteristics were not included in the analysis. ^c^ *P* values are from non-Parametric t-tests. ^d^Two-way ANOVA tests.

A total of five alleles containing 9–13 repeats were observed for the TG-*PCA3* STR. The most common TG-*PCA3* STR was the 11 repeats allele, which was significantly associated with prostate cancer risk. Prostate cancer patients had higher frequency of the 11 repeats allele (76%) compared to the control group (71%) as shown in Table 3. The 11 TG-*PCA3* STR allele was associated with a significant increase of prostate cancer risk at the allelic level (OR = 1.49; 95% CI 1.11–1.99; *P* = 0.008), while the TG-*PCA3* 12 repeats allele was associated with decreased prostate cancer risk (OR = 0.74; 95% CI 0.63–0.86; *P* < 0.0001). For the genotype analysis, the 11/11 genotype was used as a reference, heterozygous 11/12 (OR = 0.80; 95% CI 0.67–0.95; *P* = 0.01) and 12/12 homozygous (OR = 0.61; 95% CI 0.44–0.83; *P* = 0.002) genotypes were associated with a significant decrease of prostate cancer risk (Table 3). Age and family-history corrected analysis showed similar results, and all significant differences were confirmed by bootstrapping analysis (Table 3).

**Table 3:** Genotype and allele associations of TG*-PCA3* STR with prostate cancer risk.

Genotype	Cases (%)	Controls (%)	OR (95% CI)^a^	p-value^a^	OR (95% CI)^b^	*P*-value^b^	*P*-value^c^	*P*-value^d^	*P*-value^e^	*P*-value^f^
10/10	2 (0.2)	3 (0.2)	—	—	—	—	—	—	—	—
10/11	2 (0.2)	1 (0.1))	—	—	—	—	—	—	—	—
10/12	3 (0.2)	0	—	—	—	—	—	—	—	—
11/9	1 (0.1)	0	—	—	—	—	—	—	—	—
11/11	680 (59)	634 (52)	Reference	—	—	—	—	—	—	—
11/12	392 (34)	461 (38)	0.80 (0.67–0.95)	0.01	0.77 (0.64–0.92)	0.005	0.008	0.001	<0.0001	0.001
11/13	2 (0.2)	0	—	—	—	—	—	—	—	—
12/12	73 (6.3)	113 (9)	0.61 (0.44–0.83)	0.002	0.58 (0.42–0.81)	0.001	0.002	0.003	0.001	0.002
12/13	1 (0.1)	1 (0.1)	—	—	—	—	—	—	—	—
**Allele**										
9	1 (0.04)	0	—	—	—	—	—	—		
10	9 (0.4)	7 (0.3)	—	—	—	—	—	—	—	—
11	1757 (76)	1730 (71)	1.49 (1.11–1.99)	0.008	1.55 (1.14–2.1)	0.006	0.015	0.015	0.012	0.017
12	542 (23)	688 (28)	0.74 (0.63–0.86)	<0.0001	0.71 (0.61–0.84)	<0.0001	0.002	0.002	<0.0001	0.001
13	3 (0.1)	1 (0.04)	—	—	—	—	—	—	—	—

Calculated using ^a^binary logistic regression, ^b^age corrected binary logistic regression, ^c^bootstrap (two-tailed), ^d^bootstrap (two-tailed) age corrected, ^e^family history corrected binary logistic regression, ^f^bootstrap (two-tailed) family history corrected. The 11/11 repeats was used as reference for genotype analysis (IBM SPSS Statistic Processor; 23). GS: Gleason score; ns: no significant: CI: confidence interval.

### TG*-PCA3* is associated with prostate cancer aggressiveness

A case only analysis was performed to analyze the association of TG-*PCA3* STR with prostate cancer aggressiveness based on a patient’s Gleason score. The TG-*PCA3* 11 repeats allele had a higher frequency in patients with Gleason score ≥8 (OR = 2.00; 95% CI 1.06–3.76; *P* = 0.031) (Table 4). Similar results were obtained in an age-adjusted analysis (OR = 2.33; 95% CI 1.16–4.67; *P* = 0.017, Table 4), suggesting the 11 TG-*PCA3* STR repeats’ association with aggressiveness is independent of age. Bootstrapping and age corrected-bootstrapping analysis confirmed the significant differences observed (Table 4).

**Table 4:** Genotype and allele associations of TG*-PCA3* STR with Gleason scores.

Genotype	GS <8	GS ≥8	OR (95% CI)^a^	*P*-value^a^	OR (95% CI)^b^	*P*-value^b^	*P*-value^c^	*P*-value^d^	*P*-value^e^	*P*-value^f^
10/10	2 (0.2)	0	—	—	—	—	—	—	—	—
10/11	2 (0.2)	0	—	—	—	—	—	—	—	—
10/12	2 (0.2)	1 (0.7)	—	—	—	—	—	—	—	—
11/9	0	1 (0.7)	—	—	—	—	—	—	—	—
11/11	534 (58)	86 (59)	Reference	—	—	—	—	—	—	—
11/12	309 (34)	52 (36)	—	ns	—	—	—	—	—	—
11/13	2 (0.2)	0	—	—	—	—	—	—	—	—
12/12	64 (7)	5 (3)	—	ns	—	—	—	—	—	—
12/13	1 (0.1)	0	—	—	—	—	—	—	—	—
**Allele**										
9	0	1 (0.3)	—	—	—	—	—	—	—	—
10	8 (0. 4)	1 (0.3)	—	—	—	—	—	—	—	—
11	1381 (75)	225 (78)	2.00 (1.06–3.76)	0.031	2.33 (1.16–4.67)	0.01	0.017.	0.01.	0.02	0.007
12	440 (24)	63 (22)	—	ns	—	—	—	—	—	—
13	3 (0.2)	0	—	—	—	—	—	—	—	—

Calculated using ^a^binary logistic regression, ^b^age corrected binary logistic regression, ^c^bootstrap (two-tailed), ^d^bootstrap (two-tailed) age corrected, ^e^family history corrected binary logistic regression, ^f^bootstrap (two-tailed) family history corrected. The 11/11 repeats was used as reference for genotype analysis (IBM SPSS Statistic Processor; 23). GS: Gleason score; ns: no significant: CI: confidence interval.

**Figure 5:**
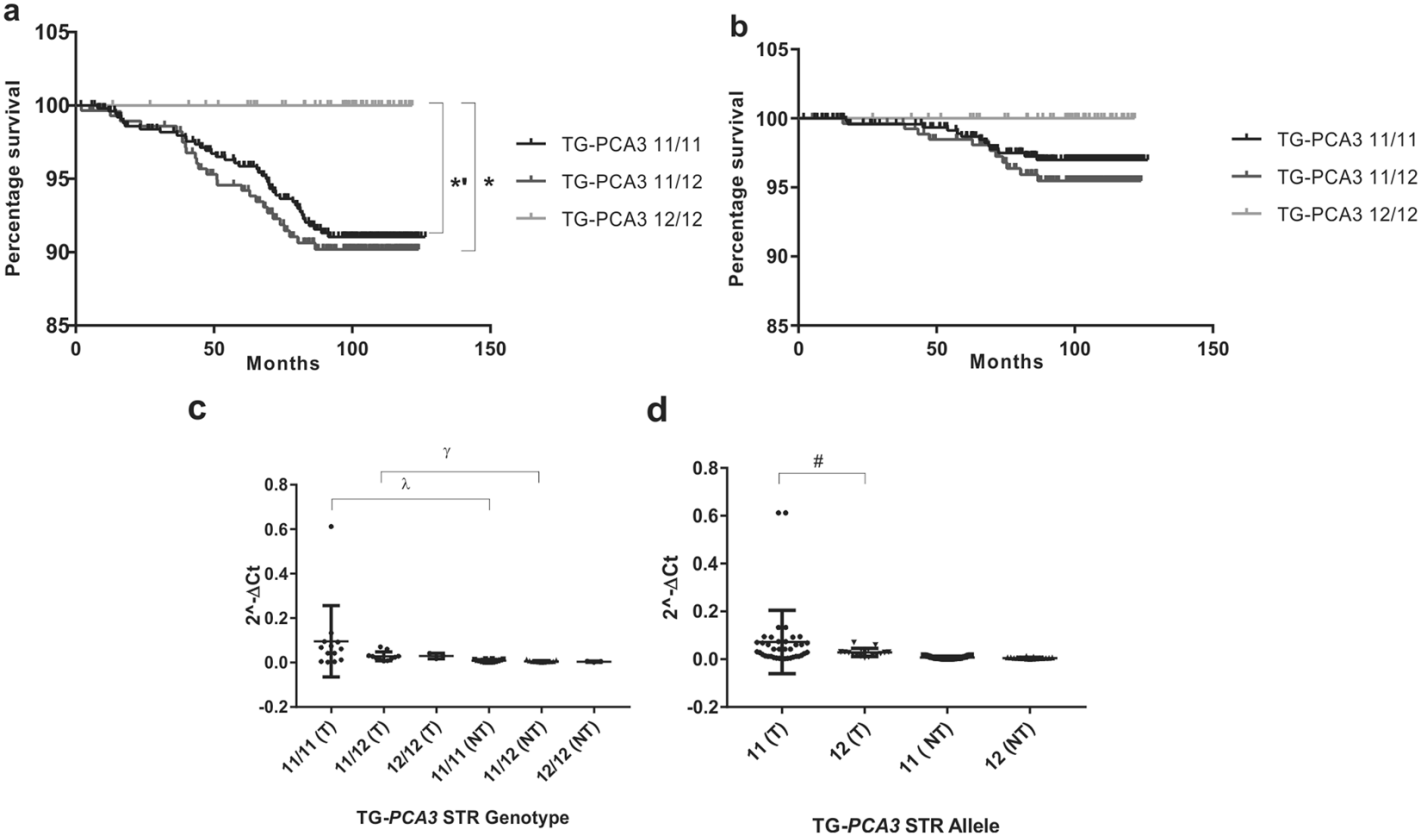
Patients’ mortality data for the 11 and 12 repeats TG-*PCA3* genotypes. **(a**) Overall mortality (n = 845; *p = 0.045; *p = 0.032). **(b**) prostate cancer specific mortality (n = 802). 2^−∆Ct^ analysis from tumor (T) and adjacent non-tumor (NT) tissue. **(c**) Genotype expression (^λ^ *P* = 0.0031; ^ɣ^ *P* = 0.0013) and **(d**), Allele expression analysis (^#^ *P* = 0.0496). *P* values calculated with: Kaplan-Meier (Log-rank (Mantel-Cox)) (**a**,**b**); and Kolmogorov-Smirnov (**c**,**d**) tests.

Survival analysis showed patients with the 12/12 genotype had a significantly lower mortality when compared to patients with the 11/12 (HR = 0.31; 95% CI 0.11–0.91; *P* = 0.032) and 11/11 (HR = 0.33; 95% CI 0.11–0.97; *P* = 0.048) genotypes (Fig. 5a). No significant differences were observed between TG-*PCA3* genotypes and prostate cancer specific mortality (Fig. 5b).

### TG-*PCA3* STR genotype correlates with *PCA3* mRNA level

The mean *PCA3* expression was slightly higher in tumors with the 11/11 TG-*PCA3* STR genotype compared to tumors with the 11/12 and 12/12 genotypes (Fig. 5c). However, statistical significance was not achieved. *PCA3* was significantly higher in tumors with 11-repeats allele compared to tumors with the 12-repeats allele (*P* = 0.049; Fig. 5d), suggesting that TG-*PCA3* STR may regulate *PCA3* expression in prostate tumor. Over-expression of *PCA3* was significantly higher in tumor compared to the adjacent non-tumor tissue in patients with the 11/11 (*P* = 0.0322) and 11/12 (*P* = 0.0013) genotypes (Fig. 5c). Significance was not achieved in patients with the 12/12 genotype, most likely due to the low number of tissue samples available with this genotype.

## Discussion

There is a growing interest in STRs as modulators of disease^12^, with recent concerted efforts made in characterizing STRs in the human genome using high-throughput sequencing approaches^3,24^. Here, we highlight that STRs are an underappreciated source of genetic variation for genetic epidemiology studies. For example, STRs are the fifth most common genetic variation, and our conservative estimate indicates that 120,806 STRs may be accurately genotyped for risk association studies. We also reveal that the human genome selects for certain types of STRs (di-nucleotides with low GC nucleotide composition), which supports the view that many STRs are indeed functional given that conservation is a measure of functionality. Notably, Willems *et al*., recently catalogued the polymorphic status of all STRs within the human genome^3^. We have incorporated the data from the Willems *et al*. study into this study to identify polymorphic STRs that are readily expressed in prostate (cancer) cells as we consider that STR expansion within genes that are critical to prostate cancer progression may further attenuate or enhance this progression, given that STR expansion affects over 40 diseases^8^.

The GWAS approach typically involves screening the genome for SNPs that correlate with prostate cancer risk, then following up with fine mapping of the risk region, and concluding with functional validation of the causal SNP. In this study, we propose an alternative approach whereby putative functional STRs are first identified prior to performing large-scale case control studies due to the cost and time constraints of high-throughput genotyping of STRs. As a first step in this approach, this study focused on STRs that are located within potentially critical prostate cancer genes by selecting for genes that are either differentially expressed in prostate tumors compared to adjacent non-malignant prostate cells, and/or genes that are regulated by androgens and/or therapeutic anti-androgens which might inform of prostate cancer genes that are involved in treatment resistance.

Consistent with our earlier observation that the human genome selects for certain types of STRs, our RNAseq analysis reveals that prostate cells also selects against genes that express certain types of STRs, notably long STRs with a consequent high number of repeats. Our RNAseq analysis also reveals that genes with penta- and hexa-nucleotide repeats are more highly expressed compared to tetra-, tri, and di-nucleotide STRs. Interestingly, we found that STRs that are expressed in LNCaP prostate cancer cells are predominantly found in the 3′UTR and coding sequence, whereas STRs that are expressed in the clinical prostate samples are predominantly located within intronic and intergenic regions. A recent study reveals that castrate resistant prostate cancer cells express high levels of intronic DNA that possibly results from inefficient/deregulated splicing that is caused by global increases in transcription^25^. Thus, it is possible that the higher proportion of STRs within introns in ours and Ren *et al*.’s clinical samples reflect this hypothesis. Importantly, STR expansion within introns can also impact biology by forming secondary DNA structures, and/or forming toxic RNA/DNA hybrids^26^. However, it is unclear why there is a high proportion of STR expression in intergenic DNA, and a low proportion in gene regions for the clinical prostate samples. It is possible that these differences may be the result of the library preparation for RNAseq, whereby the Ren *et al*. study used poly-A selected RNA and random hexamer priming in the RT, while the LNCaP RNAseq used ribosomal RNA depleted RNA and poly-A priming, and the eight clinical samples used ribosomal RNA depleted RNA and random hexamer priming. Nevertheless, any biological role of these differentially expressed STRs are likely to be mediated at a non-protein coding capacity.

Using a novel metric, we were able to identify eight STRs from prostate cancer RNAseq data sets that are most readily and/or most consistently differentially expressed in prostate tumors compared to adjacent non-malignant prostate cells. Importantly for six of the eight candidates, (*TRIB1*, *PCA3*, *GRHL2*, *ACTG2*, *GSN*, and *MXI1)* we were able to confirm the differential expression of the genes that harbor these STRs using a large dataset of prostate cancers compared to adjacent non-malignant cells. Apart from the TG-*PCA3* STR, none of the other seven STRs are located within genes that have strong links to prostate cancer, thus emphasizing their novelty. We also assessed the androgen and anti-androgen regulation of these five candidate polymorphic STRs given the importance of the AR signaling pathway and therapeutic targeting in prostate cancer^27^. Notably, only GAAA-*GRHL2*, TTTTG-*TRIB1*, TG-*PCA3*, and CAAAA-*MXI1* are expressed in LNCaP cells, and TTTTG-*TRIB1*, TG-*PCA3*, and CAAAA-*MXI1* are regulated by androgens and/or therapeutic anti-androgens. Thus, collectively, we propose that TTTTG-*TRIB1* and TG-*PCA3* are excellent genetic markers to prioritize for large-scale case-control studies as they are (i) consistently differentially expressed in prostate tumors compared to adjacent non-cancer cells, (ii) polymorphic, and (iii) are within genes regulated by (anti)-androgens (Table 1).

As a proof of concept, we analyzed the *PCA3* STR in our large case-control cohort and found it to be significantly associated with prostate cancer risk, Gleason score, and all-cause mortality. *PCA3* is a long non-protein coding RNA (lncRNA) that is gaining interest as a prostate cancer urine biomarker to complement the current PSA blood test^28^. However, the role of this lncRNA and its mechanism of action in prostate cancer are still unclear. A recent study has shown a potential method by which *PCA3* increases prostate cell proliferation and prostate tumor growth in a xenograft model^29^. The *PCA3* lncRNA binds to the *PRUNE2* pre-mRNA in prostate cancer cell lines (LNCaP and PC3), generating a double stranded RNA molecule and therefore regulating the expression of *PRUNE2* at both mRNA and protein levels^29^.

Our analysis of a large cohort of prostate cancer patients and controls found the 11 repeats allele of TG-*PCA3* STR to be significantly associated with prostate cancer risk.

A significant difference was observed in the frequency of TG-*PCA3* STR alleles, when patients were grouped by their Gleason score. The TG-*PCA3* STR 11 repeats allele had higher frequency in patients with a Gleason score of ≥8. Further, patients that carried at least one copy of this risk allele had a significant poorer prognosis in our overall survival analysis when compared to 12/12 homozygous patients. However, no significant association was observed with prostate cancer specific mortality. This could be due to the limited power of this study resulting from having a limited number of events of prostate cancer specific deaths in our cohort. A survival analysis in a larger cohort will confirm the trend observed in this study, where the TG-*PCA3* STR 11 repeats allele is associated with lower survival.

Significant over-expression of *PCA3* was observed in tumors compared to the adjacent non-tumor tissue in patients with the 11/11 and 11/12 TG-*PCA3* genotypes. TG-*PCA3* STR alleles correlated with *PCA3* expression, where a significantly higher expression was observed in tumors with the 11 repeats allele when compared to the tumors with the 12 repeats allele. These results suggest that one of the mechanisms by which the TG-*PCA3* STR 11 repeats allele is associated with a higher risk of prostate cancer is by regulating *PCA3* expression. The TG-*PCA3* STR may deregulate *PCA3* expression by modifying the 3′UTR seed region where microRNAs bind for mRNA regulation^30^. Indeed, longer seed regions are evolutionary conserved compared to shorter ones, suggesting they have been evolutionary selected for being more effective in mRNA regulation^31^. Subsequently, we hypothesize that the association of the shorter 11 repeats allele with prostate cancer risk and higher *PCA3* expression may be due to the weaker seed region for potential miRNAs in comparison to the 12 repeats allele

Notably, a TAAA STR in the *PCA3* promoter was recently found to correlate with prostate cancer risk in Chinese men^32^. It would be interesting to determine if this STR interacts synergistically or is in a linkage disequilibrium with the TG-*PCA3* STR that in turn accounts for the increased prostate cancer risk observed in this, and the Zhou *et al*. study. There is also a possibility that the STR in *PCA3* could simply be a surrogate for other known risk-alleles. However, our expression correlation analysis indicate towards the functional role of the TG-*PCA3* STR.

One of the weakness of the study could be the contamination of the control group with patients with clinically insignificant or not-yet-detected prostate cancer. Unfortunately, a long term follow-up of the controls was out of the scope of the current study. Nevertheless, univariate logistic regression analysis adjusted for age and family-history resulted in similar risk estimates for association of the STR genotypes with prostate cancer risk as was the initial unadjusted analysis.

In the current study, rather than undertaking a study in a two stage case-control design with a smaller sample set, we nominated to conduct a meta-analysis in a large sample set to provide robust risk estimates. We compensated for the lack of a replication study by conducting bootstrap analysis. To the best of our knowledge, ours is the largest study of STRs in prostate cancer, although additional genetic association studies in an independent and larger cohort is warranted. Overall, we envisage that future genetic epidemiology studies could benefit from adopting a similar approach to identify other (prostate) cancer related STRs that have predictive/prognostic value.

## Methods

### Prostate cancer patients and healthy controls

Formalin-fixed paraffin embedded (FFPE) blocks from prostate tumors and their adjacent non-malignant cells were obtained from the Australian Prostate Cancer BioResource (APCB) tumor bank. Tissue blocks containing the tumor cells were serially sectioned (20 μm sections) and transferred to glass slides. Slides were stained with methyl green and the tumor areas were marked and the Gleason grade scored by a pathologist (Supplementary Table S5). Marked areas were then manually dissected under a microscope using a sterile injection needle (size 0.65 × 25 mm).

For the genetic association study, the patient cohort with prostate cancer (N = 1,153) included 133 men recruited via collaborations with urologists, 345 men from the QLD node of the APCB, 675 men recruited in collaboration with The Cancer Council Queensland, the ProsCan study^33–35^. Details of age, family history and ethnicity and blood samples for DNA extraction, pathology reports and medical records, including Gleason scores and PSA levels to document the clinical characteristics of the disease were collected.

Cancer-free control participants (N = 1,210) included 538 age- and postal code-matched healthy male controls recruited through the Electoral Roll to complement participants in the ProsCan study, and 672 age-selected male controls recruited through the Australian Red Cross Blood Services. All controls were required to complete a detailed questionnaire on age, family history of cancer (up to 2° relatives) and other health-related factors such as BMI and smoking history. Most of the case and control samples had European background. All methods were carried out in accordance with relevant guidelines and regulations, and all experimental protocols were approved by QUT’s Human Ethics Committee (Ethics’ Approval number: 1000001171), the Australian Red Cross Services (Ethics’ Approval number: 2004#17) and Cancer Council Queensland (Ethics’ Approval number: 3629 H). All patients provided informed written consent to participate in our prostate cancer genetic studies.

### Characterization of STRs in the human genome

A flow-diagram of the bioinformatics research strategy is detailed in Supplementary Figure S5. Essentially, STRs were defined from the ‘Simple_repeats’ category from the RepeatMasker library (hg19.fa.out, Repeat Library 20120124^36^). Custom Perl scripts were used to characterize the number, length and GC nucleotide composition of STRs in the human genome from the RepeatMasker library. Polymorphic STRs within the RepeatMasker library were determined by screening against the lobSTR program^37^ predicted STRs that were carried out on the Phase 1, 1000 Genomes Project datasets (1000Genomes_Phase_1.vcf.gz^38^) from the Willems *et al*. study^3^ using a custom Perl script. Essentially, lobSTR predicted (non)-polymorphic STRs were identified within the RepeatMasker library if the chromosomal coordinates overlapped, and if the motifs (including reverse, and reverse complement motifs) matched (Supplementary Figure S5). This was carried out to filter out STRs that are less likely to be bona-fide as they are only predicted and validated by one program/study.

### Androgen and anti-androgen treatment of prostate cancer cell line

The AR positive, LNCaP, prostate cancer cell line (validated with a 100% match to the ATCC database by DDC Medical, Ohio, USA) was treated with androgen (10 nM DHT, Sigma-Aldrich, Sydney, Australia), or therapeutic anti-androgens (10uM bicalutamide, 10 uM enzalutamide, Selleckchem.com, Waterloo, Australia) for 24 h as described previously^39^.

### RNA isolation and RNA sequencing (RNAseq)

RNA from tissue samples was extracted using the miRNeasy FFPE kit (QIAGEN, Chadstone, Australia) and RNA from LNCaP cell lines (androgen and anti-androgen treated) was extracted using the RNeasy Mini Kit (QIAGEN, Chadstone, Australia). RNAseq was performed on androgen and anti-androgen treated LNCaP cell line RNA from eight clinical prostate tumors and their adjacent non-malignant cells through the Australian Genome Research Facility (AGRF). Ribosomal depleted RNA was paired-end sequenced on the Illumina HiSeq platform using 100 nucleotide read lengths, and using the Illumina TruSeq strand-specific protocol (Life Technologies, Mulgrave, Australia). RNAseq reads that map to multiple regions of the genome were filtered out using the HI:i: variable in SAM files, as well as filtering out reads that don’t have a proper pair, are chimeric alignments, or which have PCR or optical duplicates using the FLAG scores in the SAM files. The average number of RNAseq reads for each sample was 23,331,172 (Supplementary Table S5).

### Determining STR alleles and STR expression from RNAseq data

The expression of STRs from our LNCaP prostate cancer RNAseq dataset^39^, our eight clinical prostate cancer RNAseq datasets and the Ren *et al*. RNAseq dataset of 14 clinical prostate cancers and their corresponding non-cancer prostate RNA^40^, were determined using the lobSTR program^37^. lobSTR identifies and quantifies STR expression in high-throughput sequencing data such as RNAseq datasets. This program was benchmarked against capillary electrophoresis (gold standard) STR calls with at least 89.5% concordance^3^. Default parameters using RNAseq FASTQ files were used for the lobSTR analysis. The number of STR reads was determined from the ‘ALLREADS’ format field and the number of reads were normalized against total mapped RNAseq reads to determine the FPKM value. STR alleles that had less than 10 reads were excluded from the analysis.

### Differential gene expression analysis of the candidate STRs harboring genes

Transcriptome analysis of differential expression of the eight genes that harbored the candidate STRs were assessed in our clinical samples, in the Ren *et al*. dataset of 14 prostate cancer samples and their matched adjacent non-malignant cells^40^, and from the Taylor *et al*. study of 29 non-cancer and 131 prostate cancers^23^ using a student’s t-test and the log_2_ median-centered intensity values. The RNAseq data sets were analyzed by mapping RNAseq reads using Tophat2^41^ (hg19 assembly), and differentially expressed genes were determined using the edgeR program^42^.

### RT-qPCR validation

RNA was reverse transcribed (RT) using superscript III (Life Technologies) as described before^43^. Quantitative PCR (RT-qPCR) was carried out using SYBR Green mastermix (Life Technologies) using primers detailed in Supplementary Table S2. Gene expression was determined using the delta-delta CT method, using *18S* as the house-keeping gene. Data is represented as the mean plus standard error from six independent experiments and the student’s t-test was performed. For the expression-genotype correlation analysis in clinical samples, the RT-qPCR results were analysed using the delta CT method and the geomean of *HPRT1* and *RPL32* as housekeeping control genes.

### DNA extraction and STR genotyping

Ten milliliters of venous blood were collected in EDTA as a source of peripheral blood leukocytes. Genomic DNA was extracted and purified according to established protocols^35,44^ by using the QIAamp DNA Mini Kit (Qiagen, Hilden, Germany). Five of the eight candidate STRs (TAAA-*ACTG2*, GAAA-*GRHL2*, TTTTG-*TRIB1*, TG-*PCA3*, CAAAA-*MXI1*, GAAA-*PTGIS*, and TTTTTG-*PRUNE2*) were genotyped in 40 individuals using the Applied Biosystems 3500 Genetic Analyser. Briefly, 40 cycles of PCR were carried out using the Multiplex PCR kit (QIAGEN) and fluorescently labelled primers (Supplementary Table S6) according to the manufacturer’s instructions. STR allele sizes were determined using GeneMapper v.5.0 (Life Technologies). Homozygous PCR products were sequenced (AGRF) and used as positive controls for the GeneMapper Software analysis. Similarly, the prostate cancer patient and control cohorts were genotyped for the *PCA3* dinucleotide repeat using the Applied Biosystems 3500 Genetic Analyser.

### Statistical analysis

The values for age are reported as mean ± SD. Statistical analysis of age was performed by the unpaired t-test (GraphPad Prism 7.00). BMI and age were analyzed using a nonparametric, unpaired t-test. For other parameters such as smoking, drinking and marital status, their frequencies for both cases and controls were calculated and analyzed using a paired, non-parametric t-test. For parameters where only two pairs of values were available, such as vasectomy and family history, a two-way ANOVA test was used. A chi-square test using a confidence level of 0.05 was used to determine whether STRs are within the Hardy-Weinberg equilibrium. Association of TG-*PCA3* STR was analysed for prostate cancer risk and disease aggressiveness using univariate binary logistic regression (IBM SPSS Statistics; 23.0) where the dependant variable was the case-control status or the Gleason score category. A *P* < 0.05 was considered significant, and OR and 95% confidence interval (CI) were estimated. To confirm that the values obtained were not age related or prostate cancer family history associated, the results were age- and prostate cancer family history-corrected, using allele/genotype as the categorical covariate, age or prostate cancer family history as the second covariate and case-control status or Gleason score as the dependent variable. To test the association with prostate cancer aggressiveness, patients were grouped as follows: GS <8 = less aggressive, GS ≥8 = aggressive disease. For all genotype association analysis, the most common homozygous 11/11 genotype was used as a reference. Random sampling with replacement tests was carried out using the bootstrapping analysis (IBM SPSS Statistics 23.0) using a seed value of 1,000 samples 1,000,000 times.

Unless specified, statistical analysis was performed considering only the 11/11, 11/12 and 12/12 genotypes of the *PCA3* STR. The rest of the genotypes were not included in the analysis due to their low frequency. Survival analysis was conducted using the Log-rank (Mantel-Cox) test from GraphPad Prism 7.00 and data was plotted.

The *PCA3* STR genotype correlation with *PCA3* mRNA level RT-qPCR was performed on 28 patients’ RNA from tumor tissues and their adjacent non-malignant cells, which were selected based on their TG-*PCA3* STR genotypes. The genotype-expression correlation was determined using the non-parametric Kolmogorov-Smirnov test and plotted using GraphPad Prism 7.00.

## Electronic supplementary material

Supplemental material
