# Supplementary material for: A microsatellite repeat in PCA3 long non-coding RNA is associated with prostate cancer risk and aggressiveness
Source: Sci Rep. 2017 Dec 4;7:16862. doi: 10.1038/s41598-017-16700-y (PMC5715103; doi:10.1038/s41598-017-16700-y)
Supplement: Supplementary file 1 — Supplemental material [file 41598_2017_16700_MOESM1_ESM.pdf]

**A microsatellite repeat in *PCA3* long non-coding RNA is associated with prostate cancer risk and aggressiveness**

John Lai[1,2], Leire Moya[1,2], Jiyuan An[1,2], Andrea Hoffman[1,2], Srilakshmi Srinivasan[1,2], Janaththani Panchadsaram[1,2], Carina Walpole[1,2], Joanna L. Perry-Keene[3], Suzanne Chambers[4], Australian Prostate Cancer BioResource[1, 2], Melanie L. Lehman[1,2], Colleen C. Nelson[1,2], Judith A. Clements[1,2], Jyotsna Batra[1,2, *]

[1]Australian Prostate Cancer Research Centre – Queensland, Translational Research Institute, Brisbane, 4102, Australia

[2]Cancer Program, School of Biomedical Sciences, Institute of Health and Biomedical Innovation, Queensland University of Technology, Brisbane, 4102, Australia

[3]Anatomical Pathology, Pathology Queensland, Brisbane, Australia

[4]Allied Health Research, Menzies Health Institute Queensland, Griffith University, Brisbane, 4111, Australia

*corresponding author: jyotsna.batra@qut.edu.au

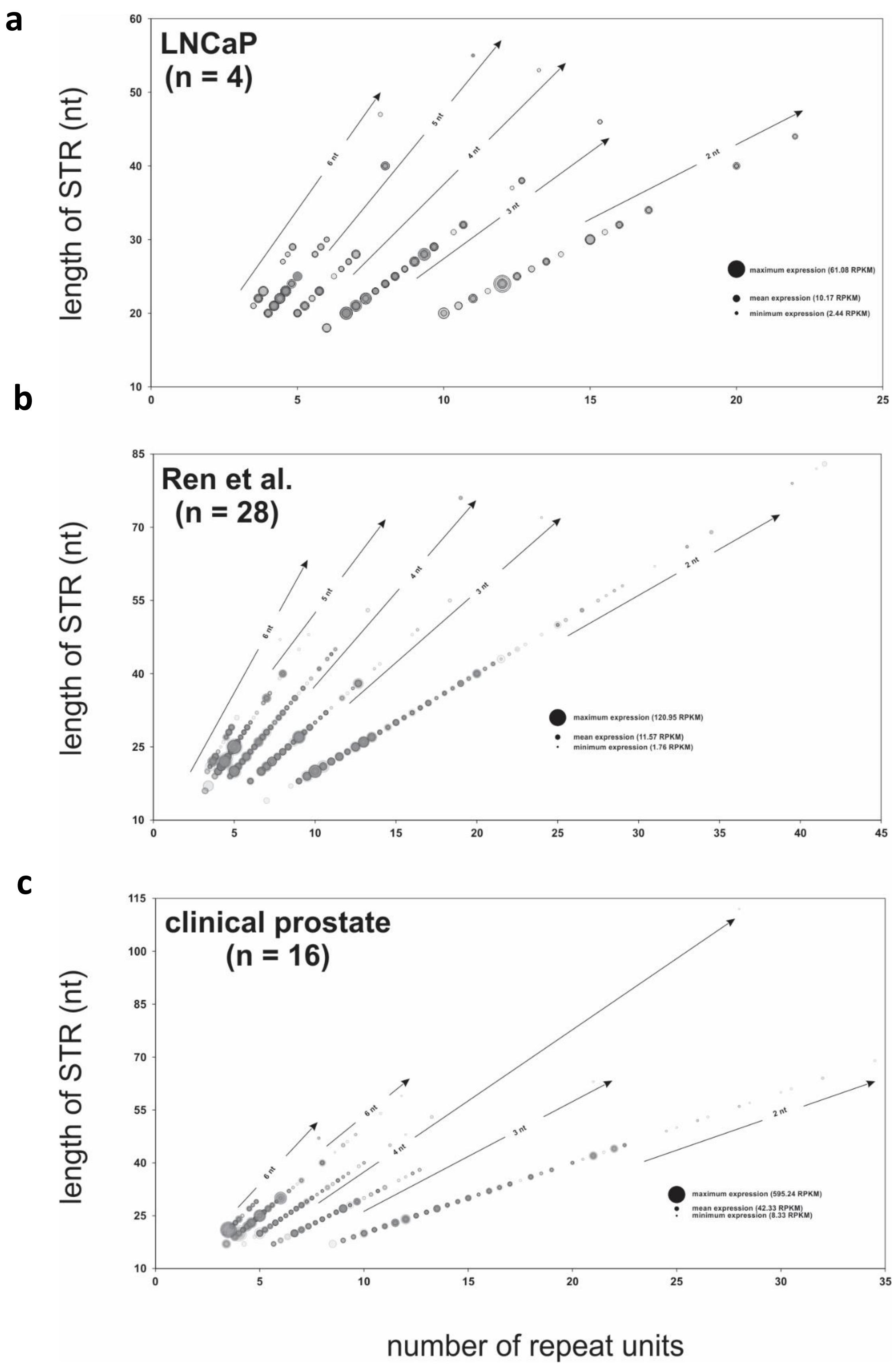


**Supplementary Figure S1.** Bubble plot of STR expression (size of bubble) relative to the length of the STR (y-axis) and the number of repeats (x-axis) for di- (2 nt), tri- (3 nt), tetra- (4 nt), penta- (5 nt), and hexa- (6 nt) nucleotide repeat STRs in **(a)** LNCaP cells, **(b)** the Ren *et al*. dataset, and **(c)** the clinical prostate samples. The number of RNAseq samples for each analysis is indicated below the dataset name.

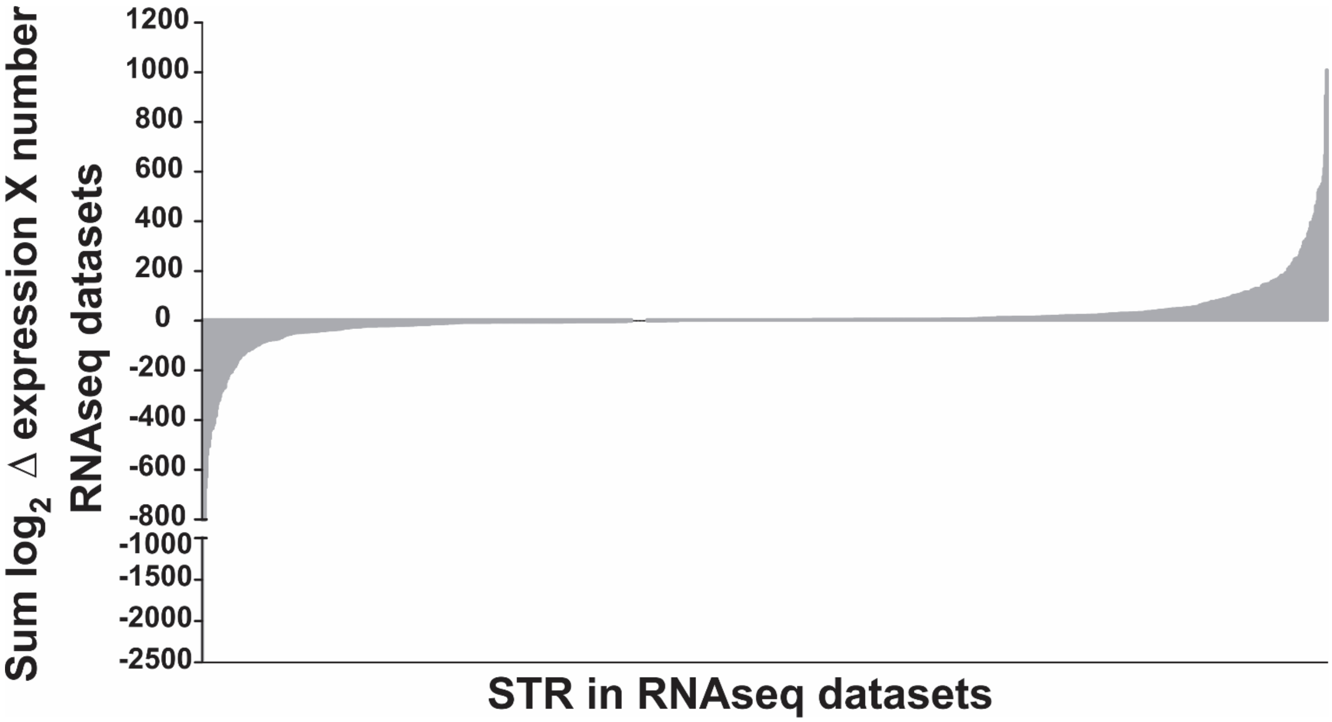


**Supplementary Figure S2**. Waterfall plot of STRs that are consistently up- or down-regulated in prostate tumors compared to their corresponding non-cancer prostate cells. Values above the line refer to STRs that are up-regulated in tumors, while below the line refer to STRs that are down-regulated in tumors.

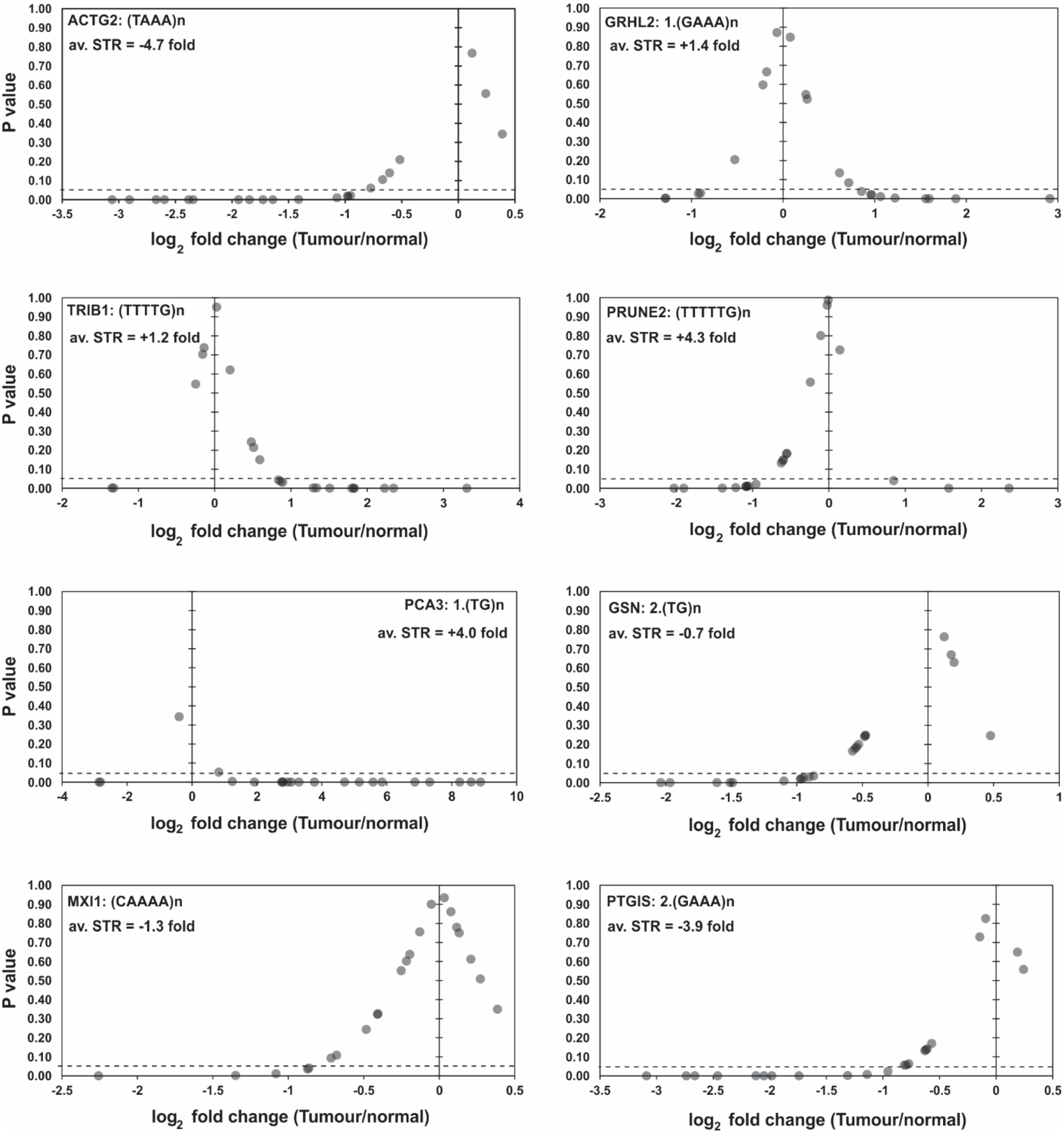


**Supplementary Figure S3. Differential expression of 8 genes harbouring STRs in prostate cancer vs non-cancerous cells.** The average **$\log_2$** fold change in expression for the corresponding STR are shown below the gene name. Differential gene expression was determined by edgeR analysis, and RNAseq reads were mapped by Tophat2 (hg19). The dotted line indicates a P value of 0.05.

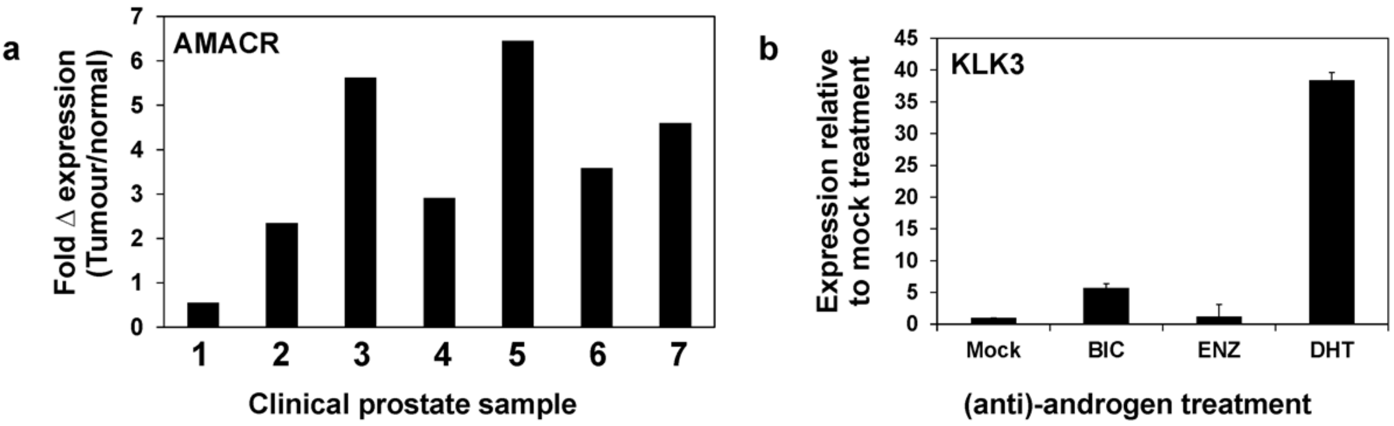


**Supplementary Figure S4**. RT-qPCR analysis of positive controls. **(a)**, The up-regulation of the AMACR prostate cancer biomarker in all clinical samples indicates that tumors and adjacent non-cancer prostate cells were appropriately extracted. **(b)**, The expression of the prototypical androgen regulated gene, KLK3, indicates that cells were appropriately treated by anti-androgens: bicalutamide (BIC), enzalutamide (ENZ) and androgen (DHT). Data is represented as the SEM from 6

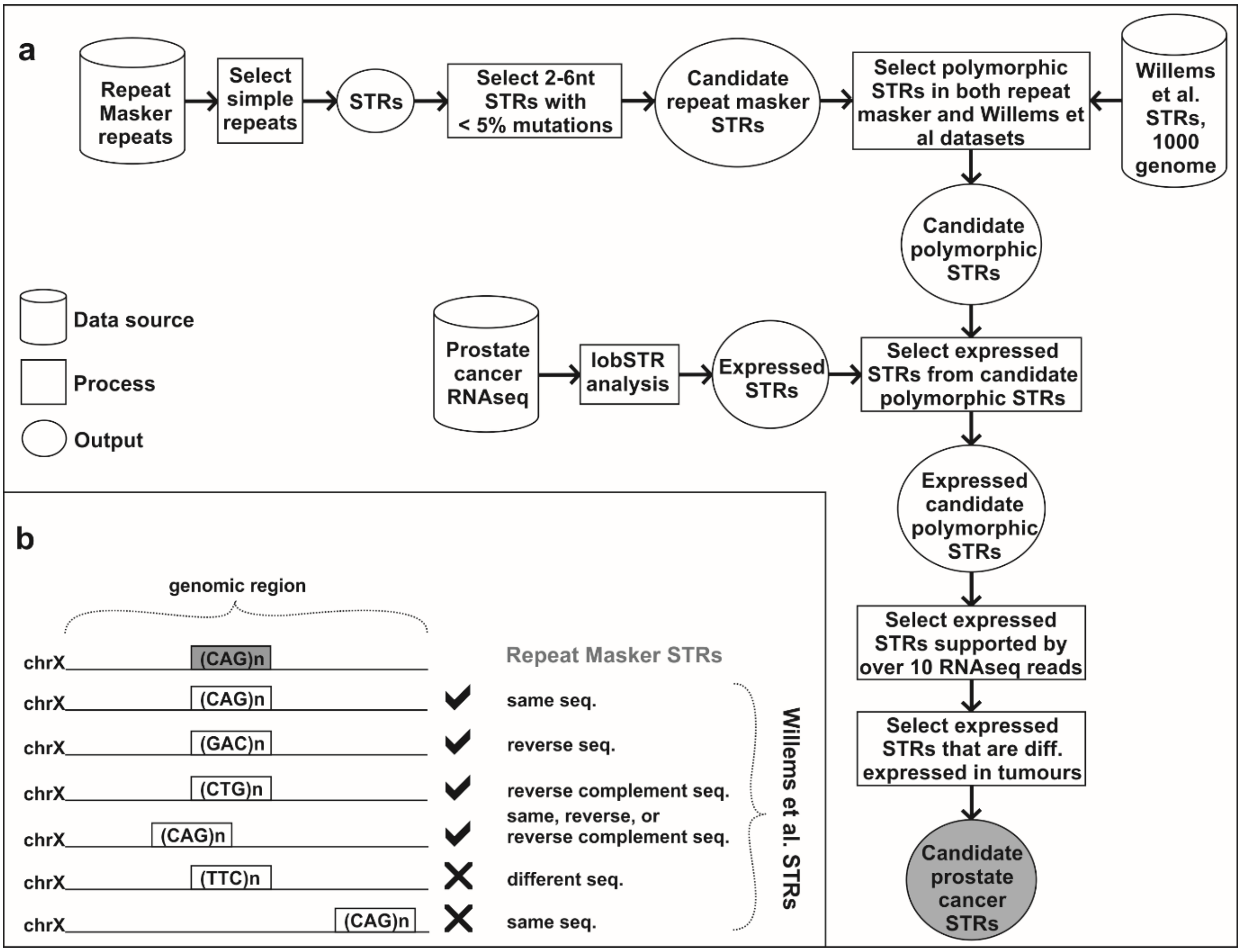


**Supplementary Figure S5. (a),** Bioinformatics workflow for identifying and characterising prostate cancer expressed STRs. (b), Algorithm used to identify STRs within the Repeat Masker track from the Willems et. al. dataset of polymorphic STRs. In this example of a chromosome X locus, if the genomic co-ordinates of STRs between the two datasets both overlap and have matching sequences (seq.), then they are considered the same STR (tick). Otherwise, they are not considered the same STR (cross).

**Supplementary Table S1**. Percentage of expressed STRs in their genomic loci from RNAseq datasets.

| | All_datasets | LNCaP | Ren_et_al | Clinical_prostate |
|---|---|---|---|---|
| **Intergenic** | 542 | 8 | 385 | 149 |
| **Promoter** | 5 | 1 | 1 | 3 |
| **5_UTR** | 42 | 12 | 17 | 13 |
| **Coding** | 62 | 31 | 18 | 13 |
| **Intron** | 875 | 17 | 300 | 558 |
| **3_UTR** | 286 | 77 | 112 | 97 |
| **total** | 1812 | 146 | 833 | 833 |

**Supplementary Table S2.** RT-qPCR primers and genotype data of the eight candidate STRs. [a]Supported by more than 10 RNAseq reads

| str_region _(hg19) | rep_mask_repeat | Primer_L | Primer_R | Product _length | Gene | Genomic_ loci | Willems_et_al_genotypes_(1000_genome) |
|---|---|---|---|---|---|---|---|
| chr2:74144316-74144336 | TAAA | TGCACTCCAGCCTGTCTCTA | TTTCCCATCTCCTGATTTCCT | 112 | ACTG2 | intron | 3.75,5.25,6,6.5,6.75,7.25,7.5,8.25 |
| chr8:102563848-102563874 | GAAA | GGTGCAAAGGAGTTTGGCTA | TAGGCTTCAATTGGGTTGGT | 117 | GRHL2 | intron | 2.75,4.5,4.75,5,5.25,5.75,6.5,7.5 |
| chr8:126450287-126450311 | TTTTG | GAATGCCGTGTATACCTCACG | CGCAGGTTATTCAGACAGACA | 139 | TRIB1 | 3'_UTR | 2.6,3,3.2,3.4,3.6,3.8,4,4.4,5,5.2 |
| chr9:79395653-79395679 | TTTTTG | CAAAAACAAGCAGCAAGAGG | CATCTCCCGATACGATGGAG | 127 | PRUNE2 | intron | 1.66667,2.66667,3.33333,3.66667,4,4.16667,4.5,5 |
| chr9:79400650-79400676 | TG | GGGTTCACTCCTGGCAATAA | GGTCAAAGAGAGAGAGGCACA | 121 | PCA3 | intergenic | 11,12,12.5,14,15,17 |
| chr9:124094978-124094997 | TG | TGCCTTTTGGAACTGTCCTT | TTTTGCAGGGCTATTTTTGG | 130 | GSN | 3'_UTR | 5.5,7,7.5,8,8.5,9,10,10.5,11,11.5,12,12.5 |
| chr10:112044843-112044867 | CAAAA | TGGGTTCATGATGCAGTCTC | CTGCCCCACTTTTTGCTAAG | 115 | MXI1 | 3'_UTR | 3.2,3.8,5 |
| chr20:48121708-48121728 | GAAA | TTTCATTCTTGCCACTGAGC | GGAAGTTACCTGGATGCCATT | 122 | PTGIS | 3'_UTR | 4,4.5,4.75,5.25,5.75,6 |
| NA | NA | ATAAGGCCACCACCAGCAAA | CCACGATATCAACTATTTGGCTTT | 111 | AMACR | NA | NA |
| NA | NA | AGTGCGAGAAGCATTCCCAAC | CCAGCAAGATCACGCTTTTGTT | 138 | KLK3 | NA | NA |
| NA | NA | GATCCATTGGAGGGCAAGTCT | CCAAGATCCAACTACGAGCTTTTT | 103 | 18S | NA | NA |

**Supplementary Table S3**. Differential analysis of genes harbouring STRs in Taylor *et al*. study.

| GEO ID | Sample Number | Patient ID | Cancer and Normal Type | Biopsy Gleason Grades |
|---|---|---|---|---|
| GSM527863 | 185 | 8 | Prostate Carcinoma | 3 + 4 |
| GSM527865 | 87 | 10 | Prostate Carcinoma | 3 + 3 |

| | | | | |
|---|---|---|---|---|
| GSM527866 | 160 | 11 | Prostate Carcinoma | 3 + 3 |
| GSM527867 | 184 | 12 | Prostate Carcinoma | 3 + 3 |
| GSM527875 | 180 | 22 | Prostate Carcinoma | 3 + 3 |
| GSM527879 | 179 | 27 | Prostate Carcinoma | 3 + 2 |
| GSM527885 | 151 | 33 | Prostate Carcinoma | 3 + 3 |
| GSM527886 | 174 | 34 | Prostate Carcinoma | 3 + 3 |
| GSM527887 | 158 | 35 | Prostate Carcinoma | 3 + 3 |
| GSM527895 | 60 | 56 | Prostate Carcinoma | 3 + 3 |
| GSM527897 | 82 | 58 | Prostate Carcinoma | 3 + 3 |
| GSM527898 | 98 | 62 | Prostate Carcinoma | 3 + 3 |
| GSM527900 | 76 | 64 | Prostate Carcinoma | 3 + 3 |
| GSM527907 | 142 | 77 | Prostate Carcinoma | 3 + 3 |
| GSM527910 | 134 | 82 | Prostate Carcinoma | 3 + 3 |
| GSM527912 | 94 | 84 | Prostate Carcinoma | 3 + 3 |
| GSM527915 | 152 | 89 | Prostate Carcinoma | 3 + 3 |
| GSM527920 | 119 | 95 | Prostate Carcinoma | 3 + 3 |
| GSM527922 | 62 | 97 | Prostate Carcinoma | 3 + 3 |
| GSM527925 | 143 | 101 | Prostate Carcinoma | 3 + 3 |
| GSM527929 | 127 | 107 | Prostate Carcinoma | 3 + 3 |
| GSM527930 | 135 | 108 | Prostate Carcinoma | 3 + 3 |
| GSM527931 | 70 | 109 | Prostate Carcinoma | 3 + 3 |
| GSM527932 | 133 | 110 | Prostate Carcinoma | 3 + 3 |
| GSM527941 | 144 | 120 | Prostate Carcinoma | 3 + 3 |
| GSM527942 | 123 | 122 | Prostate Carcinoma | 3 + 3 |
| GSM527943 | 137 | 123 | Prostate Carcinoma | 3 + 3 |
| GSM527951 | 112 | 132 | Prostate Carcinoma | 3 + 3 |
| GSM527957 | 90 | 144 | Prostate Carcinoma | 3 + 4 |
| GSM527959 | 116 | 146 | Prostate Carcinoma | 3 + 3 |
| GSM527960 | 105 | 147 | Prostate Carcinoma | 3 + 3 |
| GSM527963 | 108 | 151 | Prostate Carcinoma | 3 + 3 |
| GSM527968 | 95 | 157 | Prostate Carcinoma | 3 + 3 |
| GSM527970 | 84 | 158 | Prostate Carcinoma | 3 + 3 |
| GSM527972 | 69 | 160 | Prostate Carcinoma | 3 + 3 |
| GSM527977 | 125 | 164 | Prostate Carcinoma | 3 + 3 |
| GSM527979 | 83 | 165 | Prostate Carcinoma | 3 + 3 |
| GSM527984 | 99 | 169 | Prostate Carcinoma | 3 + 3 |
| GSM527986 | 65 | 171 | Prostate Carcinoma | 3 + 3 |
| GSM527988 | 85 | 173 | Prostate Carcinoma | 3 + 4 |
| GSM527990 | 88 | 175 | Prostate Carcinoma | 3 + 4 |
| GSM527992 | 91 | 178 | Prostate Carcinoma | 3 + 3 |
| GSM527858 | 183 | 1 | Prostate Carcinoma | 3 + 3 |
| GSM527860 | 57 | 3 | Prostate Carcinoma | 3 + 4 |
| GSM527861 | 182 | 5 | Prostate Carcinoma | 3 + 3 |
| GSM527862 | 168 | 7 | Prostate Carcinoma | 3 + 4 |
| GSM527864 | 59 | 9 | Prostate Carcinoma | 3 + 3 |
| GSM527868 | 66 | 13 | Prostate Carcinoma | 3 + 4 |
| GSM527869 | 155 | 14 | Prostate Carcinoma | 3 + 3 |
| GSM527863 | 185 | 8 | Prostate Carcinoma | 3 + 4 |
| GSM527865 | 87 | 10 | Prostate Carcinoma | 3 + 3 |
| GSM527866 | 160 | 11 | Prostate Carcinoma | 3 + 3 |
| GSM527867 | 184 | 12 | Prostate Carcinoma | 3 + 3 |
| GSM527875 | 180 | 22 | Prostate Carcinoma | 3 + 3 |
| GSM527879 | 179 | 27 | Prostate Carcinoma | 3 + 2 |
| GSM527885 | 151 | 33 | Prostate Carcinoma | 3 + 3 |
| GSM527886 | 174 | 34 | Prostate Carcinoma | 3 + 3 |
| GSM527887 | 158 | 35 | Prostate Carcinoma | 3 + 3 |
| GSM527895 | 60 | 56 | Prostate Carcinoma | 3 + 3 |
| GSM527897 | 82 | 58 | Prostate Carcinoma | 3 + 3 |
| GSM527898 | 98 | 62 | Prostate Carcinoma | 3 + 3 |
| GSM527900 | 76 | 64 | Prostate Carcinoma | 3 + 3 |
| GSM527907 | 142 | 77 | Prostate Carcinoma | 3 + 3 |
| GSM527910 | 134 | 82 | Prostate Carcinoma | 3 + 3 |
| GSM527912 | 94 | 84 | Prostate Carcinoma | 3 + 3 |
| GSM527915 | 152 | 89 | Prostate Carcinoma | 3 + 3 |
| GSM527920 | 119 | 95 | Prostate Carcinoma | 3 + 3 |

| | | | | |
|---|---|---|---|---|
| GSM527922 | 62 | 97 | Prostate Carcinoma | 3 + 3 |
| GSM527925 | 143 | 101 | Prostate Carcinoma | 3 + 3 |
| GSM527929 | 127 | 107 | Prostate Carcinoma | 3 + 3 |
| GSM527930 | 135 | 108 | Prostate Carcinoma | 3 + 3 |
| GSM527931 | 70 | 109 | Prostate Carcinoma | 3 + 3 |
| GSM527932 | 133 | 110 | Prostate Carcinoma | 3 + 3 |
| GSM527941 | 144 | 120 | Prostate Carcinoma | 3 + 3 |
| GSM527942 | 123 | 122 | Prostate Carcinoma | 3 + 3 |
| GSM527943 | 137 | 123 | Prostate Carcinoma | 3 + 3 |
| GSM527951 | 112 | 132 | Prostate Carcinoma | 3 + 3 |
| GSM527957 | 90 | 144 | Prostate Carcinoma | 3 + 4 |
| GSM527959 | 116 | 146 | Prostate Carcinoma | 3 + 3 |
| GSM527960 | 105 | 147 | Prostate Carcinoma | 3 + 3 |
| GSM527963 | 108 | 151 | Prostate Carcinoma | 3 + 3 |
| GSM527968 | 95 | 157 | Prostate Carcinoma | 3 + 3 |
| GSM527970 | 84 | 158 | Prostate Carcinoma | 3 + 3 |
| GSM527972 | 69 | 160 | Prostate Carcinoma | 3 + 3 |
| GSM527977 | 125 | 164 | Prostate Carcinoma | 3 + 3 |
| GSM527979 | 83 | 165 | Prostate Carcinoma | 3 + 3 |
| GSM527984 | 99 | 169 | Prostate Carcinoma | 3 + 3 |
| GSM527986 | 65 | 171 | Prostate Carcinoma | 3 + 3 |
| GSM527988 | 85 | 173 | Prostate Carcinoma | 3 + 4 |
| GSM527990 | 88 | 175 | Prostate Carcinoma | 3 + 4 |
| GSM527992 | 91 | 178 | Prostate Carcinoma | 3 + 3 |
| GSM527858 | 183 | 1 | Prostate Carcinoma | 3 + 3 |
| GSM527860 | 57 | 3 | Prostate Carcinoma | 3 + 4 |
| GSM527861 | 182 | 5 | Prostate Carcinoma | 3 + 3 |
| GSM527862 | 168 | 7 | Prostate Carcinoma | 3 + 4 |
| GSM527864 | 59 | 9 | Prostate Carcinoma | 3 + 3 |
| GSM527868 | 66 | 13 | Prostate Carcinoma | 3 + 4 |
| GSM527869 | 155 | 14 | Prostate Carcinoma | 3 + 3 |
| GSM527870 | 74 | 15 | Prostate Carcinoma | 3 + 3 |
| GSM527871 | 172 | 17 | Prostate Carcinoma | 3 + 4 |
| GSM527872 | 73 | 18 | Prostate Carcinoma | 3 + 3 |
| GSM527873 | 130 | 20 | Prostate Carcinoma | 3 + 4 |
| GSM527874 | 58 | 21 | Prostate Carcinoma | 4 + 3 |
| GSM527876 | 167 | 24 | Prostate Carcinoma | 3 + 4 |
| GSM527877 | 181 | 25 | Prostate Carcinoma | 4 + 3 |
| GSM527878 | 159 | 26 | Prostate Carcinoma | 3 + 3 |
| GSM527881 | 78 | 29 | Prostate Carcinoma | 3 + 3 |
| GSM527882 | 177 | 30 | Prostate Carcinoma | 3 + 3 |
| GSM527883 | 175 | 31 | Prostate Carcinoma | 3 + 5 |
| GSM527888 | 165 | 36 | Prostate Carcinoma | 3 + 3 |
| GSM527889 | 171 | 37 | Prostate Carcinoma | 3 + 3 |
| GSM527890 | 169 | 38 | Prostate Carcinoma | 5 + 4 |
| GSM527891 | 166 | 40 | Prostate Carcinoma | 3 + 3 |
| GSM527892 | 162 | 50 | Prostate Carcinoma | 3 + 4 |
| GSM527893 | 170 | 52 | Prostate Carcinoma | 3 + 3 |
| GSM527896 | 161 | 57 | Prostate Carcinoma | 3 + 3 |
| GSM527901 | 157 | 65 | Prostate Carcinoma | 3 + 3 |
| GSM527902 | 156 | 66 | Prostate Carcinoma | 3 + 4 |

| | | | | |
|---|---|---|---|---|
| GSM527903 | 55 | 68 | Prostate Carcinoma | 3 + 3 |
| GSM527904 | 148 | 73 | Prostate Carcinoma | 4 + 3 |
| GSM527905 | 164 | 74 | Prostate Carcinoma | 3 + 4 |
| GSM527906 | 114 | 75 | Prostate Carcinoma | 3 + 3 |
| GSM527908 | 163 | 80 | Prostate Carcinoma | 4 + 4 |
| GSM527909 | 153 | 81 | Prostate Carcinoma | 3 + 4 |
| GSM527911 | 154 | 83 | Prostate Carcinoma | 3 + 4 |
| GSM527913 | 139 | 86 | Prostate Carcinoma | 4 + 3 |
| GSM527914 | 128 | 87 | Prostate Carcinoma | 3 + 3 |
| GSM527916 | 110 | 90 | Prostate Carcinoma | 3 + 3 |
| GSM527918 | 106 | 93 | Prostate Carcinoma | 3 + 3 |
| GSM527919 | 149 | 94 | Prostate Carcinoma | 3 + 3 |
| GSM527923 | 63 | 99 | Prostate Carcinoma | 4 + 5 |
| GSM527924 | 92 | 100 | Prostate Carcinoma | 4 + 3 |
| GSM527926 | 145 | 103 | Prostate Carcinoma | 3 + 4 |
| GSM527927 | 101 | 104 | Prostate Carcinoma | 3 + 3 |
| GSM527928 | 75 | 105 | Prostate Carcinoma | 4 + 3 |
| GSM527933 | 115 | 111 | Prostate Carcinoma | 3 + 4 |
| GSM527935 | 138 | 113 | Prostate Carcinoma | 3 + 4 |
| GSM527936 | 93 | 114 | Prostate Carcinoma | 3 + 3 |
| GSM527937 | 77 | 115 | Prostate Carcinoma | 3 + 3 |
| GSM527938 | 147 | 117 | Prostate Carcinoma | 3 + 5 |
| GSM527939 | 103 | 118 | Prostate Carcinoma | 3 + 3 |
| GSM527940 | 107 | 119 | Prostate Carcinoma | 3 + 3 |
| GSM527944 | 126 | 124 | Prostate Carcinoma | 3 + 3 |
| GSM527945 | 141 | 125 | Prostate Carcinoma | 3 + 4 |
| GSM527946 | 121 | 126 | Prostate Carcinoma | 3 + 3 |
| GSM527947 | 102 | 127 | Prostate Carcinoma | 4 + 4 |
| GSM527948 | 140 | 128 | Prostate Carcinoma | 3 + 3 |
| GSM527949 | 129 | 129 | Prostate Carcinoma | 3 + 4 |
| GSM527952 | 80 | 133 | Prostate Carcinoma | 3 + 3 |
| GSM527953 | 64 | 134 | Prostate Carcinoma | 3 + 3 |
| GSM527954 | 120 | 135 | Prostate Carcinoma | 3 + 3 |
| GSM527955 | 72 | 136 | Prostate Carcinoma | 3 + 3 |
| GSM527956 | 96 | 141 | Prostate Carcinoma | 3 + 3 |
| GSM527958 | 111 | 145 | Prostate Carcinoma | 3 + 4 |
| GSM527961 | 136 | 149 | Prostate Carcinoma | 3 + 4 |
| GSM527962 | 118 | 150 | Prostate Carcinoma | 3 + 4 |
| GSM527965 | 109 | 155 | Prostate Carcinoma | 3 + 4 |
| GSM527966 | 67 | 156 | Prostate Carcinoma | 3 + 4 |
| GSM527973 | 86 | 161 | Prostate Carcinoma | 3 + 3 |
| GSM527974 | 68 | 162 | Prostate Carcinoma | 4 + 3 |
| GSM527975 | 104 | 163 | Prostate Carcinoma | 3 + 4 |
| GSM527980 | 79 | 167 | Prostate Carcinoma | 3 + 3 |
| GSM527982 | 113 | 168 | Prostate Carcinoma | 3 + 3 |
| GSM527985 | 122 | 170 | Prostate Carcinoma | 4 + 3 |
| GSM527989 | 100 | 174 | Prostate Carcinoma | 4 + 3 |
| GSM527859 | 132 | 2 | Prostate Carcinoma | 3 + 4 |
| GSM527880 | 178 | 28 | Prostate Carcinoma | 3 + 3 |
| GSM527894 | 173 | 54 | Prostate Carcinoma | 4 + 3 |
| GSM527899 | 56 | 63 | Prostate Carcinoma | 3 + 4 |

| | | | | |
|---|---|---|---|---|
| GSM527917 | 150 | 92 | Prostate Carcinoma | 4 + 3 |
| GSM527934 | 146 | 112 | Prostate Carcinoma | 4 + 4 |
| GSM527950 | 61 | 130 | Prostate Carcinoma | 4 + 4 |
| GSM527971 | 131 | 159 | Prostate Carcinoma | 4 + 3 |
| GSM527884 | 176 | 32 | Prostate Carcinoma | 3 + 3 |
| GSM527921 | 97 | 96 | Prostate Carcinoma | 3 + 4 |
| GSM527987 | 124 | 172 | Prostate Carcinoma | 4 + 4 |
| GSM527991 | 117 | 176 | Prostate Carcinoma | 3 + 3 |
| GSM527993 | 89 | 179 | Prostate Carcinoma | 4 + 5 |
| GSM527994 | 71 | 180 | Prostate Carcinoma | 4 + 3 |
| GSM527995 | 81 | 181 | Prostate Carcinoma | 4 + 5 |
| GSM528021 | 26 | 95 | Prostate Gland | |
| GSM528022 | 27 | 107 | Prostate Gland | |
| GSM528023 | 28 | 109 | Prostate Gland | |
| GSM528024 | 29 | 110 | Prostate Gland | |
| GSM528025 | 30 | 112 | Prostate Gland | |
| GSM528026 | 31 | 113 | Prostate Gland | |
| GSM528027 | 32 | 114 | Prostate Gland | |
| GSM528028 | 33 | 115 | Prostate Gland | |
| GSM528029 | 34 | 117 | Prostate Gland | |
| GSM528030 | 35 | 120 | Prostate Gland | |
| GSM528031 | 36 | 122 | Prostate Gland | |
| GSM528032 | 37 | 125 | Prostate Gland | |
| GSM528033 | 38 | 127 | Prostate Gland | |
| GSM528034 | 39 | 128 | Prostate Gland | |
| GSM528035 | 40 | 129 | Prostate Gland | |
| GSM528036 | 41 | 134 | Prostate Gland | |
| GSM528037 | 42 | 135 | Prostate Gland | |
| GSM528038 | 43 | 136 | Prostate Gland | |
| GSM528039 | 44 | 144 | Prostate Gland | |
| GSM528040 | 45 | 147 | Prostate Gland | |
| GSM528041 | 46 | 151 | Prostate Gland | |
| GSM528042 | 47 | 157 | Prostate Gland | |
| GSM528043 | 48 | 162 | Prostate Gland | |
| GSM528044 | 49 | 164 | Prostate Gland | |
| GSM528045 | 50 | 167 | Prostate Gland | |
| GSM528046 | 51 | 169 | Prostate Gland | |
| GSM528047 | 52 | 171 | Prostate Gland | |
| GSM528048 | 53 | 172 | Prostate Gland | |
| GSM528049 | 54 | 174 | Prostate Gland | |

**(Cont.) Supplementary Table S3**. Differential analysis of genes harbouring STRs in Taylor *et al*. study.

| GEO ID | Pathology Gleason Grades | Gleason Score (Prostate Carcinoma) | Pre-Diagnosis Biopsy PSA (ng/ml) | Pre-Treatment PSA (ng/ml) |
|---|---|---|---|---|
| GSM527863 | 3 + 3 | Gleason Score 6 | 10.4 | 10.4 |
| GSM527865 | 3 + 3 | Gleason Score 6 | 5.2 | 5.2 |
| GSM527866 | 3 + 3 | Gleason Score 6 | 2 | 6.7 |
| GSM527867 | 3 + 3 | Gleason Score 6 | 3.3 | 3.26 |
| GSM527875 | 3 + 3 | Gleason Score 6 | 5.8 | 5.8 |
| GSM527879 | 3 + 3 | Gleason Score 6 | 6.69 | 6.69 |
| GSM527885 | 3 + 3 | Gleason Score 6 | 9.6 | 14.47 |

| | | | | |
|---|---|---|---|---|
| GSM527886 | 3 + 3 | Gleason Score 6 | 5.4 | 5.4 |
| GSM527887 | 3 + 3 | Gleason Score 6 | 13.1 | 13.1 |
| GSM527895 | 3 + 3 | Gleason Score 6 | | |
| GSM527897 | 3 + 3 | Gleason Score 6 | 7.2 | 7.66 |
| GSM527898 | 3 + 3 | Gleason Score 6 | 1.09 | 1.15 |
| GSM527900 | 3 + 3 | Gleason Score 6 | 3.3 | 5.52 |
| GSM527907 | 3 + 3 | Gleason Score 6 | 2.9 | 2.77 |
| GSM527910 | 3 + 3 | Gleason Score 6 | 4.11 | 4.13 |
| GSM527912 | 3 + 3 | Gleason Score 6 | 3.1 | 3.69 |
| GSM527915 | 3 + 3 | Gleason Score 6 | 7 | 7 |
| GSM527920 | 3 + 3 | Gleason Score 6 | 4.5 | 4.5 |
| GSM527922 | 3 + 3 | Gleason Score 6 | 5.3 | 5.3 |
| GSM527925 | 3 + 3 | Gleason Score 6 | 5 | 5 |
| GSM527929 | 3 + 3 | Gleason Score 6 | 1.8 | 1.8 |
| GSM527930 | 3 + 3 | Gleason Score 6 | 4.9 | 4.9 |
| GSM527931 | 3 + 3 | Gleason Score 6 | 4.8 | 5.38 |
| GSM527932 | 3 + 3 | Gleason Score 6 | 5.7 | 5.7 |
| GSM527941 | 3 + 3 | Gleason Score 6 | 3.8 | 4.07 |
| GSM527942 | 3 + 3 | Gleason Score 6 | 4.2 | 4.2 |
| GSM527943 | 3 + 3 | Gleason Score 6 | 6.6 | 6.6 |
| GSM527951 | 3 + 3 | Gleason Score 6 | 4.4 | 5.29 |
| GSM527957 | 3 + 3 | Gleason Score 6 | 5.6 | 5.6 |
| GSM527959 | 3 + 3 | Gleason Score 6 | 3.1 | 3.1 |
| GSM527960 | 3 + 3 | Gleason Score 6 | 5.4 | 5.4 |
| GSM527963 | 3 + 3 | Gleason Score 6 | 4.5 | 4.5 |
| GSM527968 | 3 + 3 | Gleason Score 6 | 3.5 | 3.5 |
| GSM527970 | 3 + 3 | Gleason Score 6 | 4.8 | 3.2 |
| GSM527972 | 3 + 3 | Gleason Score 6 | 4 | 4 |
| GSM527977 | 3 + 3 | Gleason Score 6 | 7.67 | 7.67 |
| GSM527979 | 3 + 3 | Gleason Score 6 | 6.34 | 6.34 |
| GSM527984 | 3 + 3 | Gleason Score 6 | 6.7 | 6.7 |
| GSM527986 | | Gleason Score 6 | 8.2 | 8.2 |
| GSM527988 | 3 + 3 | Gleason Score 6 | 5.24 | 5.24 |
| GSM527990 | 3 + 3 | Gleason Score 6 | 5.6 | 5.6 |
| GSM527992 | 3 + 3 | Gleason Score 6 | 4.6 | 4.65 |
| GSM527858 | 4 + 3 | Gleason Score 7 | 132 | 43.9 |
| GSM527860 | 3 + 4 | Gleason Score 7 | 4.6 | 11.3 |
| GSM527861 | 3 + 4 | Gleason Score 7 | 8.7 | 5.9 |
| GSM527862 | 3 + 4 | Gleason Score 7 | 14 | 3.8 |
| GSM527864 | 3 + 4 | Gleason Score 7 | 14.6 | 12.9 |
| GSM527868 | 3 + 4 | Gleason Score 7 | 9.4 | 9.35 |
| GSM527869 | 3 + 4 | Gleason Score 7 | 4.2 | 2.91 |
| GSM527870 | 3 + 4 | Gleason Score 7 | 2.9 | 2.9 |
| GSM527871 | 3 + 4 | Gleason Score 7 | 9.32 | 7.65 |
| GSM527872 | 4 + 3 | Gleason Score 7 | 8.5 | 9.23 |
| GSM527873 | 3 + 4 | Gleason Score 7 | 3.8 | 3.8 |
| GSM527874 | 3 + 4 | Gleason Score 7 | 9.24 | 9.64 |
| GSM527876 | 3 + 4 | Gleason Score 7 | 14.9 | 18.41 |
| GSM527877 | 3 + 4 | Gleason Score 7 | 3.7 | 3.7 |
| GSM527878 | 3 + 4 | Gleason Score 7 | 8.6 | 9.49 |
| GSM527881 | 3 + 4 | Gleason Score 7 | 6.9 | 6.82 |

| | | | | |
|---|---|---|---|---|
| GSM527882 | 3 + 4 | Gleason Score 7 | 8.55 | 12.8 |
| GSM527883 | 4 + 3 | Gleason Score 7 | 6.3 | 6.3 |
| GSM527888 | 4 + 3 | Gleason Score 7 | 6.1 | 6.92 |
| GSM527889 | 3 + 4 | Gleason Score 7 | 5 | 5 |
| GSM527890 | 4 + 3 | Gleason Score 7 | 5.5 | 5.5 |
| GSM527891 | 3 + 4 | Gleason Score 7 | 4.6 | 4.6 |
| GSM527892 | 3 + 4 | Gleason Score 7 | 6.7 | 6.7 |
| GSM527893 | 3 + 4 | Gleason Score 7 | 12 | 12 |
| GSM527896 | 3 + 4 | Gleason Score 7 | 5 | 5.43 |
| GSM527901 | 3 + 4 | Gleason Score 7 | 4 | 5.04 |
| GSM527902 | 3 + 4 | Gleason Score 7 | 11.5 | 13.6 |
| GSM527903 | 4 + 3 | Gleason Score 7 | 8.6 | 7.62 |
| GSM527904 | 4 + 3 | Gleason Score 7 | 5 | 4.26 |
| GSM527905 | 3 + 4 | Gleason Score 7 | 4.5 | 5.11 |
| GSM527906 | 3 + 4 | Gleason Score 7 | 4.8 | 4.62 |
| GSM527908 | 4 + 3 | Gleason Score 7 | 8.3 | 10.02 |
| GSM527909 | 3 + 4 | Gleason Score 7 | 5.8 | 5.8 |
| GSM527911 | 4 + 3 | Gleason Score 7 | 8.9 | 14 |
| GSM527913 | 3 + 4 | Gleason Score 7 | 3.4 | 6.63 |
| GSM527914 | 3 + 4 | Gleason Score 7 | 4.9 | 4.9 |
| GSM527916 | 3 + 4 | Gleason Score 7 | 7.4 | 7.4 |
| GSM527918 | 3 + 4 | Gleason Score 7 | 6.74 | 4.97 |
| GSM527919 | 3 + 4 | Gleason Score 7 | 8.6 | 8.6 |
| GSM527923 | 4 + 3 | Gleason Score 7 | 12.4 | 12.47 |
| GSM527924 | 3 + 4 | Gleason Score 7 | 6.95 | 6.95 |
| GSM527926 | 3 + 4 | Gleason Score 7 | 4.5 | 4.5 |
| GSM527927 | 3 + 4 | Gleason Score 7 | 2.1 | 1.6 |
| GSM527928 | 4 + 3 | Gleason Score 7 | 3.8 | 3.8 |
| GSM527933 | 3 + 4 | Gleason Score 7 | 9.2 | 6.69 |
| GSM527935 | 3 + 4 | Gleason Score 7 | 4.2 | 4.2 |
| GSM527936 | 4 + 3 | Gleason Score 7 | 9 | 9 |
| GSM527937 | 3 + 4 | Gleason Score 7 | 5.97 | 5.97 |
| GSM527938 | 3 + 4 | Gleason Score 7 | 18.58 | 22.36 |
| GSM527939 | 3 + 4 | Gleason Score 7 | 4 | 5.08 |
| GSM527940 | 4 + 3 | Gleason Score 7 | 5.3 | 2.8 |
| GSM527944 | 3 + 4 | Gleason Score 7 | 8.93 | 10.8 |
| GSM527945 | 3 + 4 | Gleason Score 7 | 5 | 4.11 |
| GSM527946 | 3 + 4 | Gleason Score 7 | 4.39 | 3.33 |
| GSM527947 | 4 + 3 | Gleason Score 7 | 5.1 | 5.93 |
| GSM527948 | 4 + 3 | Gleason Score 7 | 2.2 | 1.48 |
| GSM527949 | 3 + 4 | Gleason Score 7 | 11.7 | 11.7 |
| GSM527952 | 3 + 4 | Gleason Score 7 | 6.98 | 6.98 |
| GSM527953 | 4 + 3 | Gleason Score 7 | 24.09 | 26.02 |
| GSM527954 | 3 + 4 | Gleason Score 7 | 6.1 | 6.1 |
| GSM527955 | 4 + 3 | Gleason Score 7 | 4.8 | 4.66 |
| GSM527956 | 3 + 4 | Gleason Score 7 | 6.55 | 5.44 |
| GSM527958 | 3 + 4 | Gleason Score 7 | 6.6 | 6.6 |
| GSM527961 | 3 + 4 | Gleason Score 7 | 16 | 20.4 |
| GSM527962 | 4 + 3 | Gleason Score 7 | 11.84 | 11.84 |
| GSM527965 | 4 + 3 | Gleason Score 7 | 11.1 | 11.1 |
| GSM527966 | 3 + 4 | Gleason Score 7 | 13.3 | 13.3 |

| | | | | |
|---|---|---|---|---|
| GSM527973 | 3 + 4 | Gleason Score 7 | 6.9 | 6.9 |
| GSM527974 | 3 + 4 | Gleason Score 7 | 6.2 | 7.11 |
| GSM527975 | 3 + 4 | Gleason Score 7 | 2.65 | 2.65 |
| GSM527980 | 3 + 4 | Gleason Score 7 | 2.5 | 2.98 |
| GSM527982 | 3 + 4 | Gleason Score 7 | 3.94 | 4.27 |
| GSM527985 | 4 + 3 | Gleason Score 7 | 9.26 | 8.97 |
| GSM527989 | 4 + 3 | Gleason Score 7 | 4.6 | 4.6 |
| GSM527859 | 5 + 3 | Gleason Score 8 | 8.1 | 43.2 |
| GSM527880 | 5 + 3 | Gleason Score 8 | 3.82 | 3.82 |
| GSM527894 | 3 + 5 | Gleason Score 8 | 31 | 39.9 |
| GSM527899 | 4 + 4 | Gleason Score 8 | 15 | 15 |
| GSM527917 | 4 + 4 | Gleason Score 8 | 5.8 | 5 |
| GSM527934 | 4 + 4 | Gleason Score 8 | 25 | 33.71 |
| GSM527950 | 4 + 4 | Gleason Score 8 | 3.6 | 2.21 |
| GSM527971 | 4 + 4 | Gleason Score 8 | 4 | 5.36 |
| GSM527884 | 4 + 5 | Gleason Score 9 | 9.6 | 16.71 |
| GSM527921 | 4 + 5 | Gleason Score 9 | 5.8 | 8.32 |
| GSM527987 | 4 + 5 | Gleason Score 9 | 17.2 | 22.82 |
| GSM527991 | 4 + 5 | Gleason Score 9 | 8.1 | 8.66 |
| GSM527993 | 4 + 5 | Gleason Score 9 | 40.24 | 46.36 |
| GSM527994 | 4 + 5 | Gleason Score 9 | 8.03 | 13.34 |
| GSM527995 | 4 + 5 | Gleason Score 9 | 27 | 27 |

| **GEO ID** | **Clinical T Stage** | **Extracapsular Extension** | **Number of Nodes Removed** | **Overall Survival Followup Time (Months)** |
|---|---|---|---|---|
| GSM527863 | T2c | Established | 7 | 149.19 |
| GSM527865 | T1c | None | 3 | 35.06 |
| GSM527866 | T1c | None | 10 | 82.17 |
| GSM527867 | T2c | None | 10 | 128.43 |
| GSM527875 | T1c | Capsular Invasion | 4 | 119.16 |
| GSM527879 | T1c | Capsular Invasion | 15 | 116.83 |
| GSM527885 | T2b | None | 17 | 69.09 |
| GSM527886 | T1c | None | 9 | 109.97 |
| GSM527887 | T2b | Established | 12 | 77.8 |
| GSM527895 | T1c | None | 1 | 25 |
| GSM527897 | T1c | None | 5 | 30.19 |
| GSM527898 | T1c | None | 5 | 42.94 |
| GSM527900 | T2b | Capsular Invasion | 10 | 24.28 |
| GSM527907 | T2b | Capsular Invasion | 9 | 61.7 |
| GSM527910 | T1c | Capsular Invasion | 7 | 58.97 |
| GSM527912 | T1c | Capsular Invasion | NA | 39.66 |
| GSM527915 | T1c | Capsular Invasion | NA | 70.18 |
| GSM527920 | T1c | None | 9 | 51.58 |
| GSM527922 | T1c | None | 3 | 1.87 |
| GSM527925 | T1c | None | 8 | 62.36 |
| GSM527929 | T2b | Capsular Invasion | 8 | 56.15 |
| GSM527930 | T3a | Established | 15 | 59.14 |
| GSM527931 | T1c | None | 14 | 13.86 |
| GSM527932 | T1c | None | NA | 58.91 |
| GSM527941 | T1c | None | 18 | 62.65 |
| GSM527942 | T1c | Capsular Invasion | NA | 52.47 |
| GSM527943 | T1c | Capsular Invasion | NA | 60.06 |

| | | | | |
|---|---|---|---|---|
| GSM527951 | T2a | Capsular Invasion | NA | 48.56 |
| GSM527957 | T1c | None | NA | 37.59 |
| GSM527959 | T1c | Capsular Invasion | NA | 50.89 |
| GSM527960 | T1c | None | 5 | 45.73 |
| GSM527963 | T1c | Capsular Invasion | NA | 47.34 |
| GSM527968 | T1c | Focal | NA | 39.85 |
| GSM527970 | T1c | Capsular Invasion | NA | 31.61 |
| GSM527972 | T1c | None | NA | 12.98 |
| GSM527977 | T1c | None | NA | 54.54 |
| GSM527979 | T1c | Capsular Invasion | NA | 30.52 |
| GSM527984 | T1c | None | NA | 42.94 |
| GSM527986 | T2a | Established | 20 | 8.84 |
| GSM527988 | T1c | None | 18 | 32.69 |
| GSM527990 | T1c | None | 6 | 36.01 |
| GSM527992 | T1c | None | 11 | 37.68 |
| GSM527858 | T2a | Established | 12 | 126.2 |
| GSM527860 | T2b | Established | 7 | 93.14 |
| GSM527861 | T1c | None | 5 | 126.1 |
| GSM527862 | T1c | None | 8 | 98.6 |
| GSM527864 | T2a | None | 20 | 85.88 |
| GSM527868 | T1c | None | 3 | 10.38 |
| GSM527869 | T1c | Capsular Invasion | NA | 76.45 |
| GSM527870 | T2c | Established | NA | 22.7 |
| GSM527871 | T2a | Focal | 8 | 104.38 |
| GSM527872 | T2a | Established | NA | 18.96 |
| GSM527873 | T2b | Established | 3 | 56.94 |
| GSM527874 | T2c | Established | NA | 61.5 |
| GSM527876 | T1c | None | 6 | 90.81 |
| GSM527877 | T2b | None | 9 | 119.26 |
| GSM527878 | T1c | None | NA | 78.16 |
| GSM527881 | T2b | Capsular Invasion | 2 | 26.68 |
| GSM527882 | T2a | Capsular Invasion | 8 | 115.09 |
| GSM527883 | T2a | Established | 3 | 112.5 |
| GSM527888 | T1c | Capsular Invasion | 5 | 85.32 |
| GSM527889 | T2a | None | 9 | 104.05 |
| GSM527890 | T2b | Capsular Invasion | 7 | 100.21 |
| GSM527891 | T2a | Capsular Invasion | 7 | 89.5 |
| GSM527892 | T2b | None | 27 | 83.85 |
| GSM527893 | T1c | Established | 12 | 102.54 |
| GSM527896 | T1c | Capsular Invasion | 9 | 82.86 |
| GSM527901 | T1c | Capsular Invasion | 7 | 77.37 |
| GSM527902 | T2b | Established | 13 | 77.11 |
| GSM527903 | T2b | Capsular Invasion | 4 | 45.54 |
| GSM527904 | T3a | Focal | 3 | 65.94 |
| GSM527905 | T1c | Capsular Invasion | 8 | 84.31 |
| GSM527906 | T2a | None | 15 | 49.35 |
| GSM527908 | T1c | Capsular Invasion | 5 | 84.27 |
| GSM527909 | T2b | Established | 12 | 73 |
| GSM527911 | T3 | Established | 13 | 74.74 |
| GSM527913 | T1c | Capsular Invasion | NA | 60.85 |
| GSM527914 | T2c | Focal | 35 | 56.84 |

| | | | | |
|---|---|---|---|---|
| GSM527916 | T1c | Established | 5 | 48.43 |
| GSM527918 | T1c | Capsular Invasion | 21 | 46.42 |
| GSM527919 | T1c | None | 18 | 66.1 |
| GSM527923 | T2c | Established | 19 | 5.78 |
| GSM527924 | T2a | Capsular Invasion | 3 | 38.21 |
| GSM527926 | T1c | Capsular Invasion | 3 | 63.71 |
| GSM527927 | T2a | Focal | NA | 43.47 |
| GSM527928 | T2c | Capsular Invasion | 13 | 23 |
| GSM527933 | T1c | Capsular Invasion | 9 | 49.84 |
| GSM527935 | T1c | Capsular Invasion | 27 | 60.45 |
| GSM527936 | T1c | Established | 5 | 38.77 |
| GSM527937 | T2a | Capsular Invasion | 11 | 26.22 |
| GSM527938 | T1c | Established | 10 | 64.13 |
| GSM527939 | T1c | None | 19 | 43.83 |
| GSM527940 | T1c | Capsular Invasion | 7 | 46.49 |
| GSM527944 | T2a | Capsular Invasion | 23 | 55.2 |
| GSM527945 | T2c | Focal | 19 | 61.37 |
| GSM527946 | T1c | Capsular Invasion | 13 | 51.85 |
| GSM527947 | T2a | Capsular Invasion | 15 | 43.53 |
| GSM527948 | T2a | None | 17 | 60.98 |
| GSM527949 | T2a | Capsular Invasion | 8 | 56.87 |
| GSM527952 | T1c | None | 11 | 28.06 |
| GSM527953 | T2a | Capsular Invasion | 18 | 8.18 |
| GSM527954 | T1c | Capsular Invasion | NA | 51.65 |
| GSM527955 | T1c | Established | NA | 16.56 |
| GSM527956 | T1c | None | 3 | 41.76 |
| GSM527958 | T1c | Capsular Invasion | 12 | 48.43 |
| GSM527961 | T2c | Established | 9 | 59.17 |
| GSM527962 | T2b | Established | 8 | 51.35 |
| GSM527965 | T2a | None | 5 | 47.61 |
| GSM527966 | T1c | Focal | 8 | 10.81 |
| GSM527973 | T1c | None | NA | 33.05 |
| GSM527974 | T1c | Capsular Invasion | 13 | 11.83 |
| GSM527975 | T1c | Established | NA | 45.37 |
| GSM527980 | T2b | None | NA | 26.84 |
| GSM527982 | T2c | None | 15 | 49.02 |
| GSM527985 | T2b | Established | 14 | 52.17 |
| GSM527989 | T1c | None | 12 | 43.3 |
| GSM527859 | T1c | Capsular Invasion | 13 | 58.02 |
| GSM527880 | T1c | Established | 6 | 116.31 |
| GSM527894 | T1c | Established | 15 | 105.14 |
| GSM527899 | T2b | Established | 9 | 74.12 |
| GSM527917 | T3a | Established | 9 | 68.08 |
| GSM527934 | T3a | Established | 16 | 63.94 |
| GSM527950 | T1c | Capsular Invasion | 13 | 61.24 |
| GSM527971 | T2a | Established | 16 | 57.3 |
| GSM527884 | T2a | Established | 9 | 114.34 |
| GSM527921 | T2a | Established | 15 | 42.38 |
| GSM527987 | T1c | Established | 7 | 53.88 |
| GSM527991 | T1c | Established | 11 | 51.02 |
| GSM527993 | T1c | Established | 48 | 36.76 |

| | | | | |
|---|---|---|---|---|
| GSM527994 | T1c | Established | 8 | 14.16 |
| GSM527995 | T1c | Established | 17 | 30.03 |

| GEO ID | Race/Ethnicity (Detailed) | Recurrence Followup Time (Months) | Recurrence Status (Detailed) | Surgical Margins | T Stage |
|---|---|---|---|---|---|
| GSM527863 | Black Non Hispanic | 149.19 | No Biochemical Recurrence | Positive | T3a |
| GSM527865 | White Non Hispanic | 35.06 | No Biochemical Recurrence | Positive | T4 |
| GSM527866 | Black Non Hispanic | 82.17 | No Biochemical Recurrence | Negative | T2c |
| GSM527867 | Black Non Hispanic | 128.43 | No Biochemical Recurrence | Negative | T2c |
| GSM527875 | White Non Hispanic | 39.95 | Biochemical Recurrence | Negative | T2c |
| GSM527879 | Black Non Hispanic | 116.83 | No Biochemical Recurrence | Negative | T2b |
| GSM527885 | Black Non Hispanic | 69.09 | No Biochemical Recurrence | Negative | T2b |
| GSM527886 | White Non Hispanic | 92.98 | Biochemical Recurrence | Negative | T2b |
| GSM527887 | White Non Hispanic | 77.8 | No Biochemical Recurrence | Negative | T3a |
| GSM527895 | White Non Hispanic | 25 | No Biochemical Recurrence | Negative | T2a |
| GSM527897 | White Non Hispanic | 30.19 | No Biochemical Recurrence | Negative | T2c |
| GSM527898 | White Non Hispanic | 42.94 | No Biochemical Recurrence | Negative | T2b |
| GSM527900 | White Non Hispanic | 24.28 | No Biochemical Recurrence | Negative | T2b |
| GSM527907 | White Non Hispanic | 61.7 | No Biochemical Recurrence | Positive | T2b |
| GSM527910 | White Non Hispanic | 58.97 | No Biochemical Recurrence | Negative | T2b |
| GSM527912 | White Non Hispanic | 39.66 | No Biochemical Recurrence | Negative | T2b |
| GSM527915 | White Non Hispanic | 70.18 | No Biochemical Recurrence | Negative | T2c |
| GSM527920 | White Non Hispanic | 51.58 | No Biochemical Recurrence | Negative | T4 |
| GSM527922 | White Non Hispanic | 1.87 | No Biochemical Recurrence | Negative | T2b |
| GSM527925 | White Non Hispanic | 62.36 | No Biochemical Recurrence | Negative | T2b |
| GSM527929 | White Non Hispanic | 56.15 | No Biochemical Recurrence | Negative | T2c |
| GSM527930 | Asian | 59.14 | No Biochemical Recurrence | Negative | T3a |
| GSM527931 | White Non Hispanic | 13.86 | No Biochemical Recurrence | Negative | T2b |
| GSM527932 | White Non Hispanic | 58.91 | No Biochemical Recurrence | Negative | T2c |
| GSM527941 | White Non Hispanic | 62.65 | No Biochemical Recurrence | Negative | T2a |
| GSM527942 | White Non Hispanic | 52.47 | No Biochemical Recurrence | Negative | T2b |
| GSM527943 | White Non Hispanic | 60.06 | No Biochemical Recurrence | Negative | T2c |
| GSM527951 | White Non Hispanic | 48.56 | No Biochemical Recurrence | Negative | T2c |
| GSM527957 | White Non Hispanic | 37.59 | No Biochemical Recurrence | Negative | T2c |
| GSM527959 | White Non Hispanic | 50.89 | No Biochemical Recurrence | Negative | T2c |
| GSM527960 | White Non Hispanic | 45.73 | No Biochemical Recurrence | Negative | T2c |
| GSM527963 | White Non Hispanic | 47.34 | No Biochemical Recurrence | Negative | T2b |
| GSM527968 | White Non Hispanic | 39.85 | No Biochemical Recurrence | Positive | T3a |
| GSM527970 | White Non Hispanic | 31.61 | No Biochemical Recurrence | Negative | T2c |

| | | | | | |
|---|---|---|---|---|---|
| GSM527972 | White Non Hispanic | 12.98 | No Biochemical Recurrence | Negative | T2a |
| GSM527977 | White Non Hispanic | 54.54 | No Biochemical Recurrence | Negative | T2b |
| GSM527979 | White Non Hispanic | 30.52 | No Biochemical Recurrence | Negative | T2b |
| GSM527984 | White Non Hispanic | 42.94 | No Biochemical Recurrence | Negative | T2b |
| GSM527986 | White Non Hispanic | 8.84 | No Biochemical Recurrence | Negative | T3a |
| GSM527988 | White Non Hispanic | 32.69 | No Biochemical Recurrence | Negative | T3b |
| GSM527990 | Black Non Hispanic | 36.01 | No Biochemical Recurrence | Negative | T2b |
| GSM527992 | White Non Hispanic | 37.68 | No Biochemical Recurrence | Negative | T2b |
| GSM527858 | Black Non Hispanic | 18.5 | Biochemical Recurrence | Negative | T3c |
| GSM527860 | Black Non Hispanic | 93.14 | No Biochemical Recurrence | Positive | T3a |
| GSM527861 | Black Non Hispanic | 126.1 | No Biochemical Recurrence | Positive | T2c |
| GSM527862 | Black Non Hispanic | 98.6 | No Biochemical Recurrence | Negative | T2c |
| GSM527864 | Black Non Hispanic | 64.76 | Biochemical Recurrence | Positive | T2c |
| GSM527868 | Black Non Hispanic | 10.38 | No Biochemical Recurrence | Positive | T3a |
| GSM527869 | White Non Hispanic | 76.45 | No Biochemical Recurrence | Positive | T2c |
| GSM527870 | Black Hispanic | 22.7 | No Biochemical Recurrence | Positive | T3a |
| GSM527871 | Black Non Hispanic | 104.38 | No Biochemical Recurrence | Negative | T3a |
| GSM527872 | White Non Hispanic | 18.96 | No Biochemical Recurrence | Positive | T3a |
| GSM527873 | White Non Hispanic | 56.94 | No Biochemical Recurrence | Negative | T3a |
| GSM527874 | White Non Hispanic | 61.5 | No Biochemical Recurrence | Negative | T3a |
| GSM527876 | Black Non Hispanic | 3.94 | Biochemical Recurrence | Negative | T2c |
| GSM527877 | White Non Hispanic | 68.04 | Biochemical Recurrence | Positive | T2c |
| GSM527878 | White Non Hispanic | 78.16 | No Biochemical Recurrence | Positive | T2a |
| GSM527881 | Black Non Hispanic | 26.68 | No Biochemical Recurrence | Negative | T2b |
| GSM527882 | White Non Hispanic | 115.09 | No Biochemical Recurrence | Negative | T2b |
| GSM527883 | White Non Hispanic | 35.35 | Biochemical Recurrence | Negative | T3a |
| GSM527888 | White Non Hispanic | 85.32 | No Biochemical Recurrence | Negative | T2a |
| GSM527889 | Black Non Hispanic | 104.05 | No Biochemical Recurrence | Negative | T2a |
| GSM527890 | White Non Hispanic | 100.21 | No Biochemical Recurrence | Negative | T2b |
| GSM527891 | White Non Hispanic | 89.5 | No Biochemical Recurrence | Negative | T2b |
| GSM527892 | White Non Hispanic | 83.85 | No Biochemical Recurrence | Negative | T2b |
| GSM527893 | Black Non Hispanic | 102.54 | No Biochemical Recurrence | Positive | T3a |
| GSM527896 | White Non Hispanic | 82.86 | No Biochemical Recurrence | Negative | T2b |
| GSM527901 | White Non Hispanic | 77.37 | No Biochemical Recurrence | Positive | T2b |
| GSM527902 | White Non Hispanic | 77.11 | No Biochemical Recurrence | Negative | T3b |
| GSM527903 | Black Non Hispanic | 45.54 | No Biochemical Recurrence | Negative | T2b |
| GSM527904 | Black Non Hispanic | 13.04 | Biochemical Recurrence | Negative | T3a |

| | | | | | |
|---|---|---|---|---|---|
| GSM527905 | White Non Hispanic | 84.31 | No Biochemical Recurrence | Negative | T2b |
| GSM527906 | White Non Hispanic | 49.35 | No Biochemical Recurrence | Negative | T2b |
| GSM527908 | White Non Hispanic | 55.39 | Biochemical Recurrence | Negative | T2b |
| GSM527909 | White Non Hispanic | 9.86 | Biochemical Recurrence | Positive | T4 |
| GSM527911 | Black Hispanic | 31.8 | Biochemical Recurrence | Positive | T3a |
| GSM527913 | Black Non Hispanic | 60.85 | No Biochemical Recurrence | Positive | T2a |
| GSM527914 | White Non Hispanic | 56.84 | No Biochemical Recurrence | Negative | T3a |
| GSM527916 | White Non Hispanic | 48.43 | No Biochemical Recurrence | Positive | T3b |
| GSM527918 | White Non Hispanic | 46.42 | No Biochemical Recurrence | Negative | T2b |
| GSM527919 | Black Non Hispanic | 66.1 | No Biochemical Recurrence | Negative | T2b |
| GSM527923 | White Non Hispanic | 5.78 | No Biochemical Recurrence | Negative | T3a |
| GSM527924 | Black Hispanic | 38.21 | No Biochemical Recurrence | Negative | T2b |
| GSM527926 | White Non Hispanic | 28.65 | Biochemical Recurrence | Negative | T2b |
| GSM527927 | Unknown | 43.47 | No Biochemical Recurrence | Negative | T2b |
| GSM527928 | White Non Hispanic | 23 | No Biochemical Recurrence | Negative | T2b |
| GSM527933 | White Non Hispanic | 49.84 | No Biochemical Recurrence | Negative | T2c |
| GSM527935 | Unknown | 60.45 | No Biochemical Recurrence | Negative | T2c |
| GSM527936 | White Non Hispanic | 38.77 | No Biochemical Recurrence | Negative | T3a |
| GSM527937 | White Non Hispanic | 26.22 | No Biochemical Recurrence | Negative | T2c |
| GSM527938 | White Non Hispanic | 18.83 | Biochemical Recurrence | Positive | T3b |
| GSM527939 | Unknown | 43.83 | No Biochemical Recurrence | Negative | T2b |
| GSM527940 | White Non Hispanic | 46.49 | No Biochemical Recurrence | Negative | T2b |
| GSM527944 | White Non Hispanic | 55.2 | No Biochemical Recurrence | Negative | T2c |
| GSM527945 | White Non Hispanic | 61.37 | No Biochemical Recurrence | Negative | T3a |
| GSM527946 | White Non Hispanic | 51.85 | No Biochemical Recurrence | Negative | T2c |
| GSM527947 | White Non Hispanic | 43.53 | No Biochemical Recurrence | Negative | T2c |
| GSM527948 | White Non Hispanic | 53.82 | Biochemical Recurrence | Positive | T2c |
| GSM527949 | White Non Hispanic | 56.87 | No Biochemical Recurrence | Negative | T2c |
| GSM527952 | White Non Hispanic | 28.06 | No Biochemical Recurrence | Negative | T2b |
| GSM527953 | Unknown | 8.18 | No Biochemical Recurrence | Positive | T2b |
| GSM527954 | White Non Hispanic | 51.65 | No Biochemical Recurrence | Negative | T2b |
| GSM527955 | White Non Hispanic | 5.72 | Biochemical Recurrence | Negative | T3a |
| GSM527956 | White Non Hispanic | 41.76 | No Biochemical Recurrence | Negative | T2a |
| GSM527958 | White Non Hispanic | 48.43 | No Biochemical Recurrence | Negative | T2c |
| GSM527961 | White Non Hispanic | 59.17 | No Biochemical Recurrence | Negative | T3b |
| GSM527962 | White Non Hispanic | 51.35 | No Biochemical Recurrence | Positive | T3a |
| GSM527965 | White Non Hispanic | 47.61 | No Biochemical Recurrence | Negative | T2b |

| | | | | | |
|---|---|---|---|---|---|
| GSM527966 | White Non Hispanic | 10.81 | No Biochemical Recurrence | Negative | T4 |
| GSM527973 | White Non Hispanic | 19.02 | Biochemical Recurrence | Negative | T2b |
| GSM527974 | White Non Hispanic | 11.83 | No Biochemical Recurrence | Negative | T2b |
| GSM527975 | White Non Hispanic | 45.37 | No Biochemical Recurrence | Negative | T3a |
| GSM527980 | White Non Hispanic | 26.84 | No Biochemical Recurrence | Negative | T2b |
| GSM527982 | White Non Hispanic | 49.02 | No Biochemical Recurrence | Negative | T2b |
| GSM527985 | White Non Hispanic | 52.17 | No Biochemical Recurrence | Negative | T3a |
| GSM527989 | White Non Hispanic | 43.3 | No Biochemical Recurrence | Negative | T2b |
| GSM527859 | Black Non Hispanic | 58.02 | No Biochemical Recurrence | Positive | T2a |
| GSM527880 | White Non Hispanic | 27.6 | Biochemical Recurrence | Positive | T3b |
| GSM527894 | White Non Hispanic | 2.1 | Biochemical Recurrence | Positive | T3c |
| GSM527899 | White Non Hispanic | 74.12 | No Biochemical Recurrence | Negative | T2b |
| GSM527917 | White Non Hispanic | 16.82 | Biochemical Recurrence | Negative | T4 |
| GSM527934 | Asian | 13.21 | Biochemical Recurrence | Negative | T3a |
| GSM527950 | White Non Hispanic | 27.86 | Biochemical Recurrence | Negative | T2c |
| GSM527971 | White Non Hispanic | 1.41 | Biochemical Recurrence | Negative | T3b |
| GSM527884 | White Non Hispanic | 3.71 | Biochemical Recurrence | Positive | T3a |
| GSM527921 | White Non Hispanic | 42.38 | No Biochemical Recurrence | Negative | T3b |
| GSM527987 | White Non Hispanic | 30.56 | Biochemical Recurrence | Positive | T4 |
| GSM527991 | Black Non Hispanic | 2.56 | Biochemical Recurrence | Positive | T3b |
| GSM527993 | White Non Hispanic | 2.92 | Biochemical Recurrence | Positive | T3b |
| GSM527994 | White Non Hispanic | 1.38 | Biochemical Recurrence | Negative | T3a |
| GSM527995 | White Non Hispanic | 30.03 | No Biochemical Recurrence | Positive | T3a |

| **GEO ID** | **log$_2$_med_centered_intensisty_TRIB1_17625** | **log$_2$_med_centered_intensisty_PCA3_26071** | **log$_2$_med_centered_intensisty_ACTG2_6185** | **log$_2$_med_centered_intensisty_GRHL2_17476** |
|---|---|---|---|---|
| GSM527863 | 2.85193 | 3.69291 | 4.40317 | 1.70772 |
| GSM527865 | 2.65111 | 2.62461 | 5.1316 | 2.10978 |
| GSM527866 | 1.85979 | 0.12591 | 5.14089 | 1.21802 |
| GSM527867 | 3.12481 | 3.35955 | 4.27266 | 2.64314 |
| GSM527875 | 1.23581 | 2.86814 | 4.70101 | 1.85856 |
| GSM527879 | 2.8494 | 3.15243 | 4.86002 | 2.29372 |
| GSM527885 | 2.10824 | 2.06209 | 4.37272 | 2.25686 |
| GSM527886 | 2.02235 | 4.2486 | 4.45327 | 2.30174 |
| GSM527887 | 2.2335 | 4.28941 | 3.93303 | 2.11517 |
| GSM527895 | 1.61269 | -0.43955 | 3.71247 | 1.38823 |
| GSM527897 | 1.72892 | 4.02118 | 4.20251 | 2.81495 |
| GSM527898 | 2.13172 | 1.85651 | 4.25698 | 1.99686 |
| GSM527900 | 2.4828 | 4.34914 | 3.42323 | 2.29468 |
| GSM527907 | 2.16969 | 3.38669 | 4.73463 | 1.13904 |
| GSM527910 | 2.86167 | 4.27053 | 4.21533 | 2.22049 |

| | | | | |
|---|---|---|---|---|
| GSM527912 | 3.18685 | 3.97462 | 2.90135 | 2.37604 |
| GSM527915 | 0.89689 | 1.23445 | 3.08652 | 2.00297 |
| GSM527920 | 2.42055 | 1.54106 | 4.80547 | 2.32317 |
| GSM527922 | 2.88768 | 2.93408 | 3.64246 | 1.54698 |
| GSM527925 | 3.11998 | 3.17274 | 4.71682 | 1.91748 |
| GSM527929 | 2.66454 | 3.50111 | 3.59176 | 1.96504 |
| GSM527930 | 2.38029 | 4.16572 | 2.27025 | 2.2281 |
| GSM527931 | 2.06771 | 3.93722 | 4.39381 | 2.50622 |
| GSM527932 | 2.11938 | 2.86132 | 3.95027 | 1.53858 |
| GSM527941 | 2.40184 | 1.87166 | 4.07316 | 2.0662 |
| GSM527942 | 1.39369 | 1.97663 | 4.18892 | 1.50864 |
| GSM527943 | 1.35756 | 3.42702 | 4.36775 | 2.38664 |
| GSM527951 | 2.41898 | 1.98616 | 4.03278 | 2.31171 |
| GSM527957 | 1.59732 | 3.73704 | 3.68035 | 2.36308 |
| GSM527959 | 1.09463 | 1.66905 | 4.59611 | 2.5907 |
| GSM527960 | 2.48789 | 0.70678 | 5.03398 | 2.31254 |
| GSM527963 | 1.25439 | 3.02731 | 3.63614 | 1.4866 |
| GSM527968 | 1.99029 | 3.61948 | 3.54722 | 1.21156 |
| GSM527970 | 2.26798 | 3.94693 | 3.69703 | 2.09892 |
| GSM527972 | 1.62131 | 3.70636 | 4.28948 | 2.14533 |
| GSM527977 | 0.45783 | 3.72421 | 3.0277 | 1.17551 |
| GSM527979 | 0.60858 | 1.56702 | 3.66448 | 0.91212 |
| GSM527984 | 1.50037 | 0.47866 | 4.49054 | 2.54148 |
| GSM527986 | 2.65156 | 4.04762 | 3.46242 | 1.98788 |
| GSM527988 | 1.52516 | 4.22379 | 4.23824 | 2.78788 |
| GSM527990 | 1.01599 | 1.67318 | 4.24455 | 1.64438 |
| GSM527992 | 1.90531 | 3.50786 | 4.31396 | 1.84198 |
| GSM527858 | 1.37992 | 2.67799 | 4.51279 | 1.48549 |
| GSM527860 | 2.1922 | 2.80913 | 3.98475 | 2.07455 |
| GSM527861 | 2.04025 | 3.83914 | 4.25199 | 2.40522 |
| GSM527862 | 2.9264 | 2.73896 | 4.52181 | 2.32266 |
| GSM527864 | 1.94803 | 3.63487 | 4.52564 | 2.52431 |
| GSM527868 | 3.10208 | 1.09597 | 3.36401 | 2.06069 |
| GSM527869 | 2.95408 | 2.81223 | 4.54399 | 2.2318 |
| GSM527870 | 2.1877 | 0.46585 | 5.55605 | 1.89143 |
| GSM527871 | 1.96704 | 3.52079 | 3.6242 | 2.04706 |
| GSM527872 | 3.147 | 3.60564 | 4.24074 | 2.44061 |
| GSM527873 | 1.75008 | 1.90144 | 4.48194 | 2.28856 |
| GSM527874 | 2.48118 | 0.5079 | 3.84723 | 1.81073 |
| GSM527876 | 2.3614 | 3.98464 | 4.06952 | 2.36236 |
| GSM527877 | 2.80131 | 1.21925 | 3.8908 | 1.55191 |
| GSM527878 | 3.01924 | 2.7305 | 4.35097 | 1.71733 |
| GSM527881 | 3.44376 | 4.26552 | 4.51842 | 2.13552 |
| GSM527882 | 3.24148 | 3.46668 | 4.68083 | 1.87216 |
| GSM527883 | 2.82271 | 2.94146 | 4.00182 | 1.33744 |
| GSM527888 | 2.56604 | 3.80586 | 4.32948 | 1.76467 |
| GSM527889 | 3.35821 | 3.6991 | 4.56518 | 2.73491 |
| GSM527890 | 2.69297 | 0.2511 | 4.12889 | 1.94711 |
| GSM527891 | 1.2277 | 0.2193 | 4.8927 | 1.34786 |
| GSM527892 | 2.25756 | 1.20966 | 3.94419 | 1.62094 |
| GSM527893 | 3.66524 | 1.24408 | 4.18385 | 2.68688 |

| | | | | |
|---|---|---|---|---|
| GSM527896 | 2.04705 | 3.74716 | 3.17827 | 1.09943 |
| GSM527901 | 1.86818 | 1.08435 | 4.62936 | 1.90234 |
| GSM527902 | 1.39437 | 3.54209 | 4.37774 | 1.81061 |
| GSM527903 | 2.59038 | 4.21543 | 4.75723 | 2.51238 |
| GSM527904 | 1.83318 | 2.70519 | 4.52567 | 2.44336 |
| GSM527905 | 1.40506 | 2.47817 | 4.13048 | 1.51148 |
| GSM527906 | 1.64309 | 2.81986 | 3.91373 | 1.98242 |
| GSM527908 | 2.54547 | 3.83582 | 3.89398 | 2.30423 |
| GSM527909 | 2.27953 | 3.79548 | 3.53891 | 2.70493 |
| GSM527911 | 2.51752 | 3.84453 | 3.81295 | 1.88464 |
| GSM527913 | 1.57279 | 3.2748 | 3.92195 | 2.70142 |
| GSM527914 | 3.27805 | 4.22646 | 3.66876 | 3.05578 |
| GSM527916 | 2.44943 | 4.11641 | 4.6394 | 1.99207 |
| GSM527918 | 2.23592 | 3.73026 | 4.51893 | 2.30324 |
| GSM527919 | 2.30468 | 1.68833 | 3.6007 | 1.53883 |
| GSM527923 | 1.83865 | 3.56138 | 4.03874 | 2.44259 |
| GSM527924 | 2.81759 | 2.42065 | 4.22246 | 1.82804 |
| GSM527926 | 2.23646 | 3.80725 | 4.35563 | 2.1382 |
| GSM527927 | 1.93604 | 2.3489 | 3.82672 | 3.11821 |
| GSM527928 | 1.63936 | 0.93864 | 4.37599 | 2.10186 |
| GSM527933 | 1.45313 | 2.2788 | 4.31867 | 1.60849 |
| GSM527935 | 1.02868 | 3.1231 | 4.29561 | 1.65518 |
| GSM527936 | 1.87086 | 3.60749 | 3.70213 | 2.05745 |
| GSM527937 | 1.35085 | 4.19906 | 2.86121 | 1.88502 |
| GSM527938 | 1.32546 | 1.89967 | 4.13447 | 1.35522 |
| GSM527939 | 1.99629 | 4.35547 | 3.68649 | 2.19237 |
| GSM527940 | 3.71051 | 0.39933 | 2.84796 | 1.22835 |
| GSM527944 | 2.25346 | 4.40486 | 3.9561 | 2.23099 |
| GSM527945 | 2.26004 | 2.70416 | 4.75361 | 1.71793 |
| GSM527946 | 3.053 | 4.0246 | 3.59747 | 1.6692 |
| GSM527947 | 0.81818 | 2.25276 | 3.93069 | 2.48126 |
| GSM527948 | 1.74076 | 2.54273 | 4.4079 | 1.82397 |
| GSM527949 | 1.65374 | 3.68877 | 3.78842 | 1.90128 |
| GSM527952 | 3.51542 | 1.58625 | 3.76833 | 2.0251 |
| GSM527953 | 0.7867 | 3.00619 | 2.92605 | 2.45425 |
| GSM527954 | 3.48475 | 2.451 | 3.72703 | 2.67529 |
| GSM527955 | 2.52466 | 3.2434 | 3.45731 | 1.35891 |
| GSM527956 | 2.69918 | 4.34258 | 3.8035 | 2.05311 |
| GSM527958 | 1.31251 | 1.88764 | 4.0739 | 1.34447 |
| GSM527961 | 1.17802 | 1.74991 | 3.28953 | 1.93342 |
| GSM527962 | 1.72882 | 1.11323 | 4.26522 | 2.25107 |
| GSM527965 | 1.27063 | 4.10114 | 1.8724 | 3.00199 |
| GSM527966 | 3.46115 | 4.00491 | 3.02856 | 2.2914 |
| GSM527973 | 1.39235 | 4.03568 | 4.0049 | 1.92336 |
| GSM527974 | 1.4849 | 4.01333 | 3.39087 | 2.00795 |
| GSM527975 | 0.72124 | 2.58103 | 3.92834 | 1.59667 |
| GSM527980 | 1.93183 | 4.04324 | 4.1326 | 2.13573 |
| GSM527982 | 2.66053 | 3.85141 | 3.80534 | 2.27957 |
| GSM527985 | 1.15906 | 2.92834 | 3.96791 | 1.1917 |
| GSM527989 | 1.35616 | 0.91084 | 3.69312 | 1.6496 |
| GSM527859 | 2.12733 | 4.36435 | 3.47977 | 2.47895 |

| | | | | |
|---|---|---|---|---|
| GSM527880 | 2.34203 | 1.61127 | 5.05364 | 1.5318 |
| GSM527894 | 1.27777 | 2.43209 | 3.32305 | 1.17384 |
| GSM527899 | 1.63136 | 2.2446 | 3.69854 | 2.72173 |
| GSM527917 | 1.55524 | 4.03659 | 3.07867 | 1.98308 |
| GSM527934 | 1.61502 | 3.2962 | 2.99842 | 2.30959 |
| GSM527950 | 0.32635 | 1.62578 | 2.65663 | 1.64997 |
| GSM527971 | 1.70012 | -0.26453 | 4.73125 | 2.0651 |
| GSM527884 | 2.94857 | 4.47622 | 2.59534 | 1.76198 |
| GSM527921 | 1.83547 | 3.97972 | 3.6304 | 2.09107 |
| GSM527987 | 2.12092 | 4.26143 | 3.34795 | 1.67232 |
| GSM527991 | 1.30567 | 0.70171 | 4.44474 | 1.1177 |
| GSM527993 | 1.1808 | 2.72121 | 2.84624 | 1.112 |
| GSM527994 | 2.01075 | 3.9867 | 1.83613 | 2.16146 |
| GSM527995 | 1.40757 | 1.80892 | 0.32978 | 0.60082 |
| GSM528021 | 0.79516 | -0.73095 | 4.01742 | 2.26811 |
| GSM528022 | 1.62843 | 0.54913 | 3.96726 | 1.99606 |
| GSM528023 | 0.86495 | 0.49778 | 5.09344 | 1.38452 |
| GSM528024 | 0.85435 | -0.21116 | 4.64546 | 2.15095 |
| GSM528025 | 1.74904 | 1.50094 | 5.11774 | 1.28128 |
| GSM528026 | 0.09898 | 0.10155 | 4.02013 | 1.9306 |
| GSM528027 | -0.06092 | 0.27396 | 4.32463 | 0.92988 |
| GSM528028 | -0.0374 | -0.10554 | 5.12601 | 1.2922 |
| GSM528029 | 0.5101 | 0.68648 | 4.43661 | 1.23688 |
| GSM528030 | 0.51912 | 0.26546 | 4.68092 | 1.04204 |
| GSM528031 | 1.78137 | 0.87159 | 4.46845 | 1.30729 |
| GSM528032 | 1.42548 | 0.23323 | 4.78454 | 0.96617 |
| GSM528033 | 0.18053 | 0.00992 | 4.51385 | 1.81601 |
| GSM528034 | 1.81631 | -0.10515 | 5.12307 | 1.13584 |
| GSM528035 | 1.36414 | 1.73371 | 3.94512 | 1.44793 |
| GSM528036 | 0.89316 | 0.2108 | 4.85506 | 1.10935 |
| GSM528037 | 1.08168 | 0.1307 | 5.23977 | 1.95738 |
| GSM528038 | 1.82004 | 0.52287 | 4.2383 | 1.87761 |
| GSM528039 | 0.80711 | 1.60121 | 4.73662 | 2.02782 |
| GSM528040 | 1.51679 | 0.97861 | 4.69623 | 1.61303 |
| GSM528041 | 1.18764 | 2.8281 | 4.70269 | 2.72798 |
| GSM528042 | 0.55266 | 1.07933 | 4.92284 | 2.36169 |
| GSM528043 | -0.02081 | -0.10127 | 5.29121 | 1.47469 |
| GSM528044 | 0.42233 | -0.49563 | 5.06005 | 0.69084 |
| GSM528045 | 1.78357 | -1.13155 | 4.85843 | 2.42264 |
| GSM528046 | 0.01763 | -0.41868 | 5.17824 | 1.84814 |
| GSM528047 | 0.64865 | 0.0867 | 5.15356 | 1.90486 |
| GSM528048 | 1.5658 | -0.57309 | 5.10134 | 1.9909 |
| GSM528049 | 0.05796 | 1.9732 | 4.86524 | 2.20782 |
| | | | | |
| **Tumor_vs_normal_analysis** | | | | |
| **av_tumour** | 2.074451908 | 2.836096412 | 3.940172672 | 1.989211145 |
| **av_normal** | 0.890477586 | 0.422836207 | 4.729801034 | 1.668983103 |
| **sd_tumour** | 0.734227609 | 1.236764307 | 0.714974028 | 0.477540494 |
| **sd_normal** | 0.653353765 | 0.878837274 | 0.410339389 | 0.51626142 |
| **log_fold_change** | 1.220079008 | 2.745735754 | -0.263520641 | 0.25322707 |
| **t-test_p-value** | 4.22662E-11 | 1.51853E-17 | 1.52849E-11 | 0.003946198 |

| GEO ID | log$_2$_med_centered_intensisty_PRUNE2_13139 | log$_2$_med_centered_intensisty_GSN | log$_2$_med_centered_intensisty_MXI1 | log$_2$_med_centered_intensisty_PTGIS |
|---|---|---|---|---|
| GSM527863 | 1.25093 | 2.96989 | 0.88696 | 4.49704 |
| GSM527865 | 2.70434 | 3.38738 | 1.35823 | 4.71773 |
| GSM527866 | 1.14304 | 2.45417 | 0.74659 | 4.45947 |
| GSM527867 | 1.13233 | 2.36852 | 0.93914 | 4.51644 |
| GSM527875 | 1.42967 | 2.48075 | 0.89871 | 4.39949 |
| GSM527879 | 2.64994 | 2.72359 | 0.95556 | 4.81349 |
| GSM527885 | 1.11874 | 2.55752 | 0.74423 | 4.54432 |
| GSM527886 | 1.18632 | 3.00416 | 1.01405 | 4.40977 |
| GSM527887 | 1.09394 | 2.81598 | 0.84195 | 4.49631 |
| GSM527895 | 0.17129 | 1.94912 | 0.16881 | 4.68784 |
| GSM527897 | 2.5579 | 2.3536 | 0.88821 | 4.72238 |
| GSM527898 | 1.72809 | 2.41347 | 0.89469 | 4.65492 |
| GSM527900 | 2.13168 | 1.38061 | 0.86713 | 4.57224 |
| GSM527907 | 1.93981 | 2.46607 | 0.97351 | 4.45002 |
| GSM527910 | 1.98285 | 2.05159 | 0.87606 | 4.60059 |
| GSM527912 | 1.22889 | 2.20635 | 1.09861 | 4.54465 |
| GSM527915 | 1.65961 | 1.17883 | 0.2095 | 4.49856 |
| GSM527920 | 2.44686 | 2.58222 | 0.92059 | 4.67608 |
| GSM527922 | 0.9789 | 2.15642 | 0.78496 | 4.39807 |
| GSM527925 | 1.9877 | 2.64939 | 1.11679 | 4.63571 |
| GSM527929 | 1.17975 | 1.74038 | 0.66307 | 4.3719 |
| GSM527930 | 0.38487 | 1.38179 | 0.70043 | 4.48198 |
| GSM527931 | 1.45895 | 2.41328 | 0.63984 | 4.64783 |
| GSM527932 | 1.11275 | 1.80775 | 0.63402 | 4.31154 |
| GSM527941 | 1.07513 | 1.76715 | 0.36952 | 4.48232 |
| GSM527942 | 1.24016 | 2.23736 | 0.58466 | 4.50613 |
| GSM527943 | 1.44651 | 2.10474 | 0.76536 | 4.56634 |
| GSM527951 | 1.41463 | 2.41375 | 0.66911 | 4.46122 |
| GSM527957 | 0.60412 | 2.03398 | 1.09692 | 4.28942 |
| GSM527959 | 1.20359 | 2.08243 | 0.67306 | 4.41478 |
| GSM527960 | 2.5657 | 2.83719 | 1.08964 | 4.62796 |
| GSM527963 | 0.65885 | 2.22765 | 0.84056 | 4.39409 |
| GSM527968 | 0.52193 | 1.4206 | 0.74201 | 4.12451 |
| GSM527970 | 1.38599 | 1.66281 | 0.78226 | 4.45511 |
| GSM527972 | 1.36655 | 2.49936 | 0.90704 | 4.49926 |
| GSM527977 | 0.31236 | 2.45423 | 0.56089 | 4.47667 |
| GSM527979 | 1.16708 | 2.06629 | 0.99817 | 4.35068 |
| GSM527984 | 1.51079 | 2.47353 | 0.9054 | 4.40417 |
| GSM527986 | 1.47575 | 2.24198 | 0.79879 | 4.48389 |
| GSM527988 | 2.39852 | 2.40266 | 0.9195 | 4.72234 |
| GSM527990 | 0.79588 | 1.97252 | 0.69301 | 4.54437 |
| GSM527992 | 1.28969 | 2.08758 | 0.59863 | 4.65055 |
| GSM527858 | 1.44086 | 2.80932 | 0.92322 | 4.58678 |
| GSM527860 | 0.63544 | 2.1821 | 0.43494 | 4.3601 |
| GSM527861 | 1.39212 | 2.2477 | 0.88306 | 4.69625 |
| GSM527862 | 1.59494 | 2.23883 | 0.86308 | 4.49333 |
| GSM527864 | 1.47654 | 2.82471 | 0.72939 | 4.68438 |
| GSM527868 | 1.45402 | 2.09154 | 0.50093 | 4.45872 |
| GSM527869 | 1.85891 | 2.50261 | 0.93491 | 4.53011 |

| | | | | |
|---|---|---|---|---|
| GSM527870 | 2.67431 | 3.44056 | 0.88252 | 4.72703 |
| GSM527871 | 0.80769 | 2.16764 | 0.58837 | 4.51348 |
| GSM527872 | 1.71258 | 2.77944 | 1.0184 | 4.59423 |
| GSM527873 | 1.28826 | 2.66368 | 0.88886 | 4.54389 |
| GSM527874 | 3.28891 | 2.79542 | 0.66042 | 4.49741 |
| GSM527876 | 1.13459 | 1.8143 | 0.48055 | 4.64911 |
| GSM527877 | 1.68325 | 2.18423 | 0.9968 | 4.49472 |
| GSM527878 | 1.35602 | 2.68614 | 0.78726 | 4.67137 |
| GSM527881 | 1.85316 | 2.54347 | 0.73608 | 4.56824 |
| GSM527882 | 0.97803 | 2.88563 | 1.08818 | 4.3789 |
| GSM527883 | 0.80218 | 1.97865 | 0.68339 | 4.44864 |
| GSM527888 | 0.93824 | 2.85127 | 0.825 | 4.63486 |
| GSM527889 | 1.49446 | 2.19304 | 0.91742 | 4.57748 |
| GSM527890 | 1.21519 | 2.68817 | 1.01577 | 4.63123 |
| GSM527891 | 1.86795 | 1.96544 | 0.5101 | 4.69611 |
| GSM527892 | 1.8166 | 2.41726 | 0.47539 | 4.67369 |
| GSM527893 | 1.35758 | 2.25427 | 1.11366 | 4.63795 |
| GSM527896 | 0.07461 | 1.47063 | 0.14867 | 4.58253 |
| GSM527901 | 2.22521 | 2.21855 | 1.02335 | 4.69859 |
| GSM527902 | 1.5535 | 2.04256 | 0.52408 | 4.54365 |
| GSM527903 | 1.76671 | 2.89396 | 1.08229 | 4.57967 |
| GSM527904 | 1.87404 | 2.35539 | 0.76992 | 4.50279 |
| GSM527905 | 1.89648 | 2.10125 | 0.95309 | 4.43822 |
| GSM527906 | 2.56818 | 2.04515 | 0.5563 | 4.37484 |
| GSM527908 | 1.01176 | 2.48163 | 0.98802 | 4.61149 |
| GSM527909 | 1.59459 | 1.87268 | 0.84284 | 4.42629 |
| GSM527911 | 1.64564 | 2.05812 | 0.40445 | 4.60731 |
| GSM527913 | 1.74808 | 1.87852 | 1.03851 | 4.6129 |
| GSM527914 | 1.58041 | 1.51148 | 0.58726 | 4.52715 |
| GSM527916 | 1.87693 | 2.73359 | 1.18157 | 4.66868 |
| GSM527918 | 2.21225 | 2.17953 | 0.77024 | 4.70907 |
| GSM527919 | 0.32034 | 1.90062 | 0.62023 | 4.23097 |
| GSM527923 | 2.5313 | 2.76252 | 0.96739 | 4.68968 |
| GSM527924 | 1.78876 | 2.23928 | 0.7226 | 4.68112 |
| GSM527926 | 1.81251 | 2.27033 | 0.78442 | 4.55322 |
| GSM527927 | 1.88592 | 2.1671 | 0.63619 | 4.57834 |
| GSM527928 | 1.28802 | 2.27887 | 0.70569 | 4.62581 |
| GSM527933 | 1.37761 | 2.6979 | 0.70167 | 4.54706 |
| GSM527935 | 1.62689 | 2.20855 | 0.76828 | 4.51347 |
| GSM527936 | 0.71154 | 1.66856 | 0.39398 | 4.43825 |
| GSM527937 | 0.55772 | 1.35072 | 0.45815 | 4.39217 |
| GSM527938 | 0.58853 | 1.91061 | 0.66665 | 4.34537 |
| GSM527939 | 1.40432 | 1.52156 | 0.55402 | 4.57539 |
| GSM527940 | 0.12391 | 1.61483 | 0.50717 | 4.48398 |
| GSM527944 | 1.15393 | 1.42507 | 0.52992 | 4.59097 |
| GSM527945 | 2.07461 | 2.54139 | 0.79421 | 4.75327 |
| GSM527946 | 0.47259 | 2.08876 | 0.52601 | 4.55537 |
| GSM527947 | 2.31548 | 1.96924 | 0.37089 | 4.48784 |
| GSM527948 | 1.18403 | 1.88898 | 0.48287 | 4.48196 |
| GSM527949 | 0.80031 | 1.61239 | 0.63918 | 4.4034 |
| GSM527952 | 1.60324 | 1.89068 | 0.80451 | 4.4804 |

| | | | | |
|---|---|---|---|---|
| GSM527953 | 0.81447 | 1.98153 | 0.72614 | 4.48441 |
| GSM527954 | 0.1925 | 2.043 | 1.02151 | 4.42172 |
| GSM527955 | 0.04294 | 2.17661 | 0.87889 | 4.35436 |
| GSM527956 | 1.20081 | 1.989 | 0.81847 | 4.50429 |
| GSM527958 | 0.65359 | 2.10344 | 0.79554 | 4.56398 |
| GSM527961 | 1.83113 | 2.00652 | 0.65157 | 4.40159 |
| GSM527962 | 1.23292 | 2.60077 | 1.04725 | 4.54724 |
| GSM527965 | -0.89387 | 1.8381 | 0.65221 | 4.31298 |
| GSM527966 | -0.22582 | 1.48414 | 0.70306 | 4.57255 |
| GSM527973 | 2.02906 | 2.00796 | 0.66966 | 4.57673 |
| GSM527974 | 0.99549 | 2.15814 | 0.77881 | 4.40887 |
| GSM527975 | 0.72122 | 1.69949 | 0.64844 | 4.54837 |
| GSM527980 | 1.15916 | 2.01946 | 0.69227 | 4.16701 |
| GSM527982 | 0.54814 | 2.05465 | 0.70614 | 4.44931 |
| GSM527985 | 0.95935 | 2.65846 | 0.72038 | 4.63464 |
| GSM527989 | 0.82103 | 2.26001 | 0.73392 | 4.49306 |
| GSM527859 | 1.12401 | 2.37433 | 0.81213 | 4.47781 |
| GSM527880 | 2.19677 | 3.06435 | 1.02118 | 4.57684 |
| GSM527894 | 0.16473 | 1.96052 | 0.3412 | 4.52559 |
| GSM527899 | 2.75105 | 1.4155 | 0.86904 | 4.69735 |
| GSM527917 | 2.41429 | 1.82447 | 0.7974 | 4.43131 |
| GSM527934 | 1.43206 | 1.98573 | 0.76111 | 4.41577 |
| GSM527950 | 1.8319 | 1.17584 | 0.39584 | 4.62188 |
| GSM527971 | 1.43913 | 2.76056 | 0.98577 | 4.38067 |
| GSM527884 | 1.60789 | 2.09734 | 0.94054 | 4.27354 |
| GSM527921 | 1.74167 | 1.94654 | 1.05419 | 4.58805 |
| GSM527987 | 0.20203 | 1.90424 | 0.4927 | 4.56521 |
| GSM527991 | 1.65887 | 3.15123 | 0.74909 | 4.4626 |
| GSM527993 | -0.43181 | 1.71931 | 0.15377 | 4.40395 |
| GSM527994 | -0.54949 | 0.7789 | 0.35586 | 4.58175 |
| GSM527995 | -0.82354 | 1.10924 | 0.88271 | 4.19425 |
| GSM528021 | 1.60614 | 2.48853 | 0.66827 | 4.62222 |
| GSM528022 | 2.02402 | 2.04769 | 0.81214 | 4.49177 |
| GSM528023 | 1.94118 | 3.3513 | 0.86705 | 4.5284 |
| GSM528024 | 1.83284 | 2.14931 | 0.9445 | 4.60834 |
| GSM528025 | 2.18037 | 3.48877 | 1.24562 | 4.50771 |
| GSM528026 | 2.04655 | 2.08499 | 0.7399 | 4.37527 |
| GSM528027 | 0.97189 | 2.75948 | 0.92651 | 4.45668 |
| GSM528028 | 2.55369 | 3.43538 | 1.19692 | 4.59184 |
| GSM528029 | 1.30329 | 2.52481 | 1.05112 | 4.39068 |
| GSM528030 | 1.63066 | 2.57405 | 1.04453 | 4.33244 |
| GSM528031 | 1.26865 | 2.72363 | 0.94146 | 4.23315 |
| GSM528032 | 1.33381 | 2.63416 | 0.84289 | 4.31042 |
| GSM528033 | 1.74826 | 2.13466 | 0.78856 | 4.4488 |
| GSM528034 | 2.21243 | 3.6375 | 1.33545 | 4.53336 |
| GSM528035 | 0.72834 | 1.92373 | 0.76584 | 4.24983 |
| GSM528036 | 2.22138 | 2.90149 | 1.16915 | 4.59051 |
| GSM528037 | 2.50116 | 3.31382 | 1.46029 | 4.54491 |
| GSM528038 | 1.36478 | 2.44745 | 1.03192 | 3.98158 |
| GSM528039 | 1.85153 | 2.55469 | 1.2894 | 4.4529 |
| GSM528040 | 2.0337 | 2.48168 | 1.03573 | 4.46425 |

| | | | | |
|---|---|---|---|---|
| GSM528041 | 2.31531 | 2.39802 | 0.92218 | 4.67453 |
| GSM528042 | 2.51678 | 2.4358 | 0.99172 | 4.56611 |
| GSM528043 | 2.66179 | 3.36258 | 1.25703 | 4.48303 |
| GSM528044 | 2.48149 | 3.64155 | 1.23235 | 4.56094 |
| GSM528045 | 2.1555 | 2.5213 | 1.32031 | 4.67518 |
| GSM528046 | 2.23608 | 3.11075 | 1.0588 | 4.58972 |
| GSM528047 | 2.58002 | 3.33713 | 1.71655 | 4.70102 |
| GSM528048 | 2.05464 | 2.72375 | 0.88597 | 4.65965 |
| GSM528049 | 2.52045 | 2.65442 | 0.82429 | 4.63436 |
| | | | | |
| **Tumour_vs_normal_analysis** | | | | |
| **av_tumour** | 1.322945344 | 2.182323969 | 0.756605191 | 4.520909084 |
| **av_normal** | 1.961266552 | 2.753186897 | 1.047118966 | 4.491710345 |
| **sd_tumour** | 0.747715346 | 0.472747478 | 0.224888837 | 0.125779021 |
| **sd_normal** | 0.511347058 | 0.505632542 | 0.241625126 | 0.159123288 |
| **log_fold_change** | -0.568032164 | -0.335237261 | -0.46881278 | 0.009348009 |
| **t-test_p-value** | 7.76276E-07 | 1.99196E-06 | 6.19945E-07 | 0.360495094 |

## Supplementary Table S4: Genotypes of STRS in 40 patients

| **Sample** | **STR** | allele_1 | allele_2 | **STR** | allele_1 | allele_2 | **STR** | allele_1 | allele_2 | **STR** | allele_1 | allele_2 | **STR** | allele_1 | allele_2 | **STR** | allele_1 | allele_2 | **STR** | allele_1 | allele_2 |
|---|---|---|---|---|---|---|---|---|---|---|---|---|---|---|---|---|---|---|---|---|---|
| 1 | GAAA-GRHL2 | 5 | 5 | CAAAA-MXI1 | 4 | 5 | TAAA-ACTG2 | 5 | 6 | TTTTG-TRIB1 | 3 | 4 | TG-PCA3 | 11 | 12 | GAAA-PTGIS | 5 | 5 | TTTTTG-PRUNE2 | 5 | 5 |
| 2 | GAAA-GRHL2 | 5 | 5 | CAAAA-MXI1 | 4 | 4 | TAAA-ACTG2 | 6 | 6 | TTTTG-TRIB1 | 3 | 4 | TG-PCA3 | 11 | 11 | GAAA-PTGIS | 5 | 5 | TTTTTG-PRUNE2 | 5 | 5 |
| 3 | GAAA-GRHL2 | 5 | 5 | CAAAA-MXI1 | 4 | 5 | TAAA-ACTG2 | 5 | 5 | TTTTG-TRIB1 | 3 | 4 | TG-PCA3 | 11 | 11 | GAAA-PTGIS | 5 | 5 | TTTTTG-PRUNE2 | 5 | 5 |
| 4 | GAAA-GRHL2 | 5 | 5 | CAAAA-MXI1 | 4 | 5 | TAAA-ACTG2 | 5 | 6 | TTTTG-TRIB1 | 4 | 4 | TG-PCA3 | 12 | 12 | GAAA-PTGIS | 5 | 5 | TTTTTG-PRUNE2 | 5 | 5 |
| 5 | GAAA-GRHL2 | 5 | 5 | CAAAA-MXI1 | 5 | 5 | TAAA-ACTG2 | 6 | 6 | TTTTG-TRIB1 | 4 | 4 | TG-PCA3 | 11 | 11 | GAAA-PTGIS | 5 | 5 | TTTTTG-PRUNE2 | 5 | 5 |
| 6 | GAAA-GRHL2 | 5 | 5 | CAAAA-MXI1 | 4 | 5 | TAAA-ACTG2 | 5 | 6 | TTTTG-TRIB1 | 4 | 4 | TG-PCA3 | 12 | 12 | GAAA-PTGIS | 5 | 5 | TTTTTG-PRUNE2 | 5 | 5 |
| 7 | GAAA-GRHL2 | 5 | 5 | CAAAA-MXI1 | 4 | 5 | TAAA-ACTG2 | 5 | 6 | TTTTG-TRIB1 | 4 | 4 | TG-PCA3 | 11 | 11 | GAAA-PTGIS | 5 | 5 | TTTTTG-PRUNE2 | 5 | 5 |
| 8 | GAAA-GRHL2 | 5 | 5 | CAAAA-MXI1 | 4 | 5 | TAAA-ACTG2 | 6 | 6 | TTTTG-TRIB1 | 4 | 4 | TG-PCA3 | 11 | 12 | GAAA-PTGIS | 5 | 5 | TTTTTG-PRUNE2 | 5 | 5 |
| 9 | GAAA-GRHL2 | 5 | 5 | CAAAA-MXI1 | 4 | 4 | TAAA-ACTG2 | 5 | 6 | TTTTG-TRIB1 | 4 | 4 | TG-PCA3 | 11 | 11 | GAAA-PTGIS | 5 | 5 | TTTTTG-PRUNE2 | 5 | 5 |
| 10 | GAAA-GRHL2 | 5 | 5 | CAAAA-MXI1 | 4 | 4 | TAAA-ACTG2 | 5 | 6 | TTTTG-TRIB1 | 4 | 4 | TG-PCA3 | 11 | 11 | GAAA-PTGIS | 5 | 5 | TTTTTG-PRUNE2 | 5 | 5 |
| 11 | GAAA-GRHL2 | 5 | 5 | CAAAA-MXI1 | 5 | 5 | TAAA-ACTG2 | 5 | 6 | TTTTG-TRIB1 | 4 | 4 | TG-PCA3 | 11 | 11 | GAAA-PTGIS | 5 | 5 | TTTTTG-PRUNE2 | 5 | 5 |
| 12 | GAAA-GRHL2 | 5 | 5 | CAAAA-MXI1 | 4 | 5 | TAAA-ACTG2 | 5 | 5 | TTTTG-TRIB1 | 3 | 4 | TG-PCA3 | 11 | 12 | GAAA-PTGIS | 5 | 5 | TTTTTG-PRUNE2 | 5 | 5 |
| 13 | GAAA-GRHL2 | 5 | 5 | CAAAA-MXI1 | 4 | 5 | TAAA-ACTG2 | 6 | 6 | TTTTG-TRIB1 | 4 | 4 | TG-PCA3 | 11 | 11 | GAAA-PTGIS | 5 | 5 | TTTTTG-PRUNE2 | 5 | 5 |
| 14 | GAAA-GRHL2 | 5 | 5 | CAAAA-MXI1 | 5 | 5 | TAAA-ACTG2 | 6 | 6 | TTTTG-TRIB1 | 3 | 4 | TG-PCA3 | 11 | 11 | GAAA-PTGIS | 5 | 5 | TTTTTG-PRUNE2 | 5 | 5 |
| 15 | GAAA-GRHL2 | 5 | 5 | CAAAA-MXI1 | 4 | 5 | TAAA-ACTG2 | 5 | 6 | TTTTG-TRIB1 | 4 | 4 | TG-PCA3 | 11 | 12 | GAAA-PTGIS | 5 | 5 | TTTTTG-PRUNE2 | 5 | 5 |
| 16 | GAAA-GRHL2 | 5 | 5 | CAAAA-MXI1 | 4 | 4 | TAAA-ACTG2 | 6 | 6 | TTTTG-TRIB1 | 4 | 4 | TG-PCA3 | 11 | 11 | GAAA-PTGIS | 5 | 5 | TTTTTG-PRUNE2 | 5 | 5 |

| | | | | | | | | | | | | | | | | | | | | | |
|---|---|---|---|---|---|---|---|---|---|---|---|---|---|---|---|---|---|---|---|---|---|
| 17 | GAAA-GRHL2 | 5 | 5 | CAAAA-MXI1 | 4 | 5 | TAAA-ACTG2 | 5 | 6 | TTTTG-TRIB1 | 4 | 4 | TG-PCA3 | 11 | 11 | GAAA-PTGIS | 5 | 5 | TTTTTG-PRUNE2 | 5 | 5 |
| 18 | GAAA-GRHL2 | 5 | 5 | CAAAA-MXI1 | 4 | 4 | TAAA-ACTG2 | 6 | 6 | TTTTG-TRIB1 | 4 | 4 | TG-PCA3 | 11 | 12 | GAAA-PTGIS | 5 | 5 | TTTTTG-PRUNE2 | 5 | 5 |
| 19 | GAAA-GRHL2 | 5 | 5 | CAAAA-MXI1 | 5 | 5 | TAAA-ACTG2 | 5 | 6 | TTTTG-TRIB1 | 4 | 4 | TG-PCA3 | 11 | 12 | GAAA-PTGIS | 5 | 5 | TTTTTG-PRUNE2 | 5 | 5 |
| 20 | GAAA-GRHL2 | 5 | 5 | CAAAA-MXI1 | 4 | 5 | TAAA-ACTG2 | 5 | 6 | TTTTG-TRIB1 | 4 | 4 | TG-PCA3 | 11 | 12 | GAAA-PTGIS | 5 | 5 | TTTTTG-PRUNE2 | 5 | 5 |
| 21 | GAAA-GRHL2 | 5 | 5 | CAAAA-MXI1 | 4 | 4 | TAAA-ACTG2 | 5 | 5 | TTTTG-TRIB1 | 3 | 4 | TG-PCA3 | 11 | 12 | GAAA-PTGIS | 5 | 5 | TTTTTG-PRUNE2 | 5 | 5 |
| 22 | GAAA-GRHL2 | 5 | 5 | CAAAA-MXI1 | 4 | 5 | TAAA-ACTG2 | 5 | 6 | TTTTG-TRIB1 | 3 | 4 | TG-PCA3 | 11 | 11 | GAAA-PTGIS | 5 | 5 | TTTTTG-PRUNE2 | 5 | 5 |
| 23 | GAAA-GRHL2 | 5 | 5 | CAAAA-MXI1 | 5 | 5 | TAAA-ACTG2 | 6 | 6 | TTTTG-TRIB1 | 3 | 4 | TG-PCA3 | 11 | 11 | GAAA-PTGIS | 5 | 5 | TTTTTG-PRUNE2 | 5 | 5 |
| 24 | GAAA-GRHL2 | 5 | 5 | CAAAA-MXI1 | 4 | 5 | TAAA-ACTG2 | 5 | 5 | TTTTG-TRIB1 | 4 | 4 | TG-PCA3 | 11 | 11 | GAAA-PTGIS | 5 | 5 | TTTTTG-PRUNE2 | 5 | 5 |
| 25 | GAAA-GRHL2 | 4 | 5 | CAAAA-MXI1 | 4 | 4 | TAAA-ACTG2 | 6 | 6 | TTTTG-TRIB1 | 3 | 4 | TG-PCA3 | 11 | 12 | GAAA-PTGIS | 5 | 5 | TTTTTG-PRUNE2 | 5 | 5 |
| 26 | GAAA-GRHL2 | 5 | 5 | CAAAA-MXI1 | 4 | 5 | TAAA-ACTG2 | 5 | 6 | TTTTG-TRIB1 | 4 | 4 | TG-PCA3 | 11 | 11 | GAAA-PTGIS | 5 | 5 | TTTTTG-PRUNE2 | 5 | 5 |
| 27 | GAAA-GRHL2 | 5 | 5 | CAAAA-MXI1 | 4 | 5 | TAAA-ACTG2 | 6 | 6 | TTTTG-TRIB1 | 4 | 4 | TG-PCA3 | 11 | 11 | GAAA-PTGIS | 5 | 5 | TTTTTG-PRUNE2 | 5 | 5 |
| 28 | GAAA-GRHL2 | 5 | 5 | CAAAA-MXI1 | 4 | 5 | TAAA-ACTG2 | 5 | 6 | TTTTG-TRIB1 | 4 | 4 | TG-PCA3 | 11 | 11 | GAAA-PTGIS | 5 | 5 | TTTTTG-PRUNE2 | 5 | 5 |
| 29 | GAAA-GRHL2 | 4 | 5 | CAAAA-MXI1 | 4 | 5 | TAAA-ACTG2 | 5 | 6 | TTTTG-TRIB1 | 4 | 4 | TG-PCA3 | 11 | 11 | GAAA-PTGIS | 5 | 5 | TTTTTG-PRUNE2 | 5 | 5 |
| 30 | GAAA-GRHL2 | 5 | 5 | CAAAA-MXI1 | 5 | 5 | TAAA-ACTG2 | 5 | 6 | TTTTG-TRIB1 | 4 | 4 | TG-PCA3 | 11 | 11 | GAAA-PTGIS | 5 | 5 | TTTTTG-PRUNE2 | 5 | 5 |
| 31 | GAAA-GRHL2 | 5 | 5 | CAAAA-MXI1 | 4 | 5 | TAAA-ACTG2 | 5 | 6 | TTTTG-TRIB1 | 4 | 4 | TG-PCA3 | 11 | 11 | GAAA-PTGIS | 5 | 5 | TTTTTG-PRUNE2 | 5 | 5 |
| 32 | GAAA-GRHL2 | 5 | 5 | CAAAA-MXI1 | 4 | 4 | TAAA-ACTG2 | 6 | 6 | TTTTG-TRIB1 | 3 | 3 | TG-PCA3 | 11 | 11 | GAAA-PTGIS | 5 | 5 | TTTTTG-PRUNE2 | 5 | 5 |
| 33 | GAAA-GRHL2 | 5 | 5 | CAAAA-MXI1 | 5 | 5 | TAAA-ACTG2 | 5 | 6 | TTTTG-TRIB1 | 4 | 4 | TG-PCA3 | 11 | 12 | GAAA-PTGIS | 5 | 5 | TTTTTG-PRUNE2 | 5 | 5 |
| 34 | GAAA-GRHL2 | 5 | 5 | CAAAA-MXI1 | 4 | 5 | TAAA-ACTG2 | 5 | 6 | TTTTG-TRIB1 | 4 | 4 | TG-PCA3 | 11 | 11 | GAAA-PTGIS | 5 | 5 | TTTTTG-PRUNE2 | 5 | 5 |
| 35 | GAAA-GRHL2 | 5 | 5 | CAAAA-MXI1 | 4 | 5 | TAAA-ACTG2 | 5 | 6 | TTTTG-TRIB1 | 4 | 4 | TG-PCA3 | 11 | 12 | GAAA-PTGIS | 5 | 5 | TTTTTG-PRUNE2 | 5 | 5 |
| 36 | GAAA-GRHL2 | 5 | 5 | CAAAA-MXI1 | 4 | 5 | TAAA-ACTG2 | 5 | 6 | TTTTG-TRIB1 | 4 | 4 | TG-PCA3 | 11 | 12 | GAAA-PTGIS | 5 | 5 | TTTTTG-PRUNE2 | 5 | 5 |
| 37 | GAAA-GRHL2 | 5 | 5 | CAAAA-MXI1 | 4 | 5 | TAAA-ACTG2 | 5 | 5 | TTTTG-TRIB1 | 4 | 4 | TG-PCA3 | 11 | 12 | GAAA-PTGIS | 5 | 5 | TTTTTG-PRUNE2 | 5 | 5 |
| 38 | GAAA-GRHL2 | 5 | 5 | CAAAA-MXI1 | 4 | 4 | TAAA-ACTG2 | 5 | 5 | TTTTG-TRIB1 | 4 | 4 | TG-PCA3 | 11 | 11 | GAAA-PTGIS | 5 | 5 | TTTTTG-PRUNE2 | 5 | 5 |
| 39 | GAAA-GRHL2 | 5 | 5 | CAAAA-MXI1 | 4 | 5 | TAAA-ACTG2 | 6 | 6 | TTTTG-TRIB1 | 3 | 4 | TG-PCA3 | 11 | 12 | GAAA-PTGIS | 5 | 5 | TTTTTG-PRUNE2 | 5 | 5 |
| 40 | GAAA-GRHL2 | 5 | 5 | CAAAA-MXI1 | 4 | 4 | TAAA-ACTG2 | 6 | 6 | TTTTG-TRIB1 | 4 | 4 | TG-PCA3 | 11 | 11 | GAAA-PTGIS | 5 | 5 | TTTTTG-PRUNE2 | 5 | 5 |
| positive_controls | GAAA-GRHL2 | 5 | 5 | CAAAA-MXI1 | 4 | 4 | TAAA-ACTG2 | 6 | 6 | TTTTG-TRIB1 | 4 | 4 | TG-PCA3 | 11 | 11 | GAAA-PTGIS | 5 | 5 | TTTTTG-PRUNE2 | 5 | 5 |
| | | | | | | | | | | | | | | | | | | | | | |
| alleles | 4 | 5 | | 4 | 5 | | 5 | 6 | | 3 | 4 | | 11 | 12 | | 5 | | | 5 | | |
| % alleles | 0.025 | 0.975 | | 0.5375 | 0.463 | | 0.4125 | 0.588 | | 0.15 | 0.85 | | 0.775 | 0.225 | | 1 | | | 1 | | |
| | | | | | | | | | | | | | | | | | | | | | |
| genotype | 4/4 | 4/5 | 5/5 | 4/4 | 4/5 | 5/5 | 4/4 | 4/5 | 5/5 | 4/4 | 4/5 | 5/5 | 11/11 | 11/12 | 12/12 | 5/5 | | | 5/5 | | |
| % expected genotypes | 0.000625 | 0.049 | 0.9506 | 0.28890625 | 0.497 | 0.2139 | 0.17015625 | 0.485 | 0.3452 | 0.0225 | 0.255 | 0.7225 | 0.600625 | 0.349 | 0.0506 | 1 | | | 1 | | |

| % observed genotypes | 0 | 0.05 | 0.95 | 0.25 | 0.575 | 0.175 | 0.15 | 0.525 | 0.325 | 0.025 | 0.25 | 0.725 | 0.6 | 0.35 | 0.05 | 1 | | | 1 | | |
|---|---|---|---|---|---|---|---|---|---|---|---|---|---|---|---|---|---|---|---|---|---|
| | | | | | | | | | | | | | | | | | | | | | |
| | Heterozygosity | 0.05 | | Heterozygosity | 0.575 | | Heterozygosity | 0.525 | | Heterozygosity | 0.25 | | Heterozygosity | 0.35 | | Heterozygosity | N/A | | Heterozygosity | N/A | |
| | In_HWE | yes | | In_HWE | yes | | In_HWE | yes | | In_HWE | yes | | In_HWE | yes | | In_HWE | not tested | | In_HWE | not tested | |
| | calculated-p-value | 0.87 | | calculated-p-value | 0.32 | | calculated-p-value | 0.6 | | calculated-p-value | 0.9 | | calculated-p-value | 0.98 | | calculated-p-value | not tested | | calculated-p-value | not tested | |

## Supplementary Table S5. Information on patient samples used for the RNA-seq and TR-qPCR analysis

| Sample | Tumour_site | Gleason_score | Experiment | total_RNAseq_reads_normal | total_RNAseq_reads_tumour |
|---|---|---|---|---|---|
| 1 | Posterior | 7 | RNAseq | 20939142 | 20545681 |
| 2 | Posterior | 7 | RNAseq | 23330654 | 20465177 |
| 3 | Posterior | 8 | RNAseq | 22766169 | 27295077 |
| 4 | Posterior | 7 | RNAseq | 20527285 | 19211352 |
| 5 | Anterior | 6 | RNAseq | 26555597 | 26225578 |
| 6 | Posterior | 7 | RNAseq | 23214078 | 22700738 |
| 7 | Posterior | 7 | RNAseq | 25186759 | 24103860 |
| 8 | Posterior | 7 | RNAseq | 24009218 | 26222388 |
| 1 | Posterior | 9 | RT-qPCR | N/A | N/A |
| 2 | Anterior | 9 | RT-qPCR | N/A | N/A |
| 3 | Anterior | 8 | RT-qPCR | N/A | N/A |
| 4 | Posterior | 9 | RT-qPCR | N/A | N/A |
| 5 | Posterior | 9 | RT-qPCR | N/A | N/A |
| 6 | Posterior | 9 | RT-qPCR | N/A | N/A |
| 7 | Posterior | 8 | RT-qPCR | N/A | N/A |

## Supplementary Table S6. Genotyping primers.

| str_region_(hg19) | rep_mask_repeat | dye | Primer_F | Primer_R | Product_length[a] (bp) | Gene |
|---|---|---|---|---|---|---|
| chr2:74144316-74144336 | TAAA | FAM | TCTCTAAATATAAATTCAGATAGATCCTG | TTAAAGATACCATAGTGTGACCTCC | 141 | ACTG2 |
| chr8:102563848-102563874 | GAAA | PET | GGTGCAAAGGAGTTTGGCTA | TAGGCTTCAATTGGGTTGGT | 117 | GRHL2 |
| chr8:126450287-126450311 | TTTTG | NED | GAGAAATGGCACAAAAACAGG | TTCTGTCAAGGTAATATTGCCAA | 299 | TRIB1 |
| chr9:79400650-79400676 | TG | FAM | GTGAGTGCGCTTTAGAATTTTG | CATTTACTGGAGAATAATGAGATATGG | 329 | PCA3 |
| chr10:112044843-112044867 | CAAAA | NED | TTGAAAGCACAGAGTTCTCCC | GTCCTCTGACCCTTTTGTTCA | 295 | MXI1 |

[a]According to UCSC hg19
